# MC-NODE: A Mechanism-Decomposed Neural Differential Model for PLGA Microsphere Drug Release Prediction and Attribution

**DOI:** 10.3390/ph19081227

**Published:** 2026-08-04

**Authors:** Zi’an Tang, Hui Li, Tianfu Li, Feng Xue

**Affiliations:** 1 Department of Pharmaceutics, UCL School of Pharmacy, University College London, London WC1N 1AX, UK; 2 Graduate School of Frontier Sciences, The University of Tokyo, Kashiwa 277-8561, Japan; 3 Graduate School of Information Science and Technology, The University of Tokyo, Tokyo 113-8656, Japan; 4 Graduate School of Information, Production and Systems, Waseda University, Kitakyushu 808-0135, Japan

**Keywords:** PLGA microspheres, drug release prediction, long-acting injectables, formulation design, neural differential equations, mechanism attribution, machine learning

## Abstract

**Background/Objectives**: Poly(lactide-co-glycolide) (PLGA) microspheres support long-acting drug delivery, but their release profiles are difficult to predict because burst release, diffusion, polymer degradation, and formulation-dependent effects interact across multiple time scales. This study aimed to develop a continuous-time model that combines accurate release prediction with physically admissible trajectories and release-component attribution. **Methods**: MC-NODE encodes ten drug, polymer, and formulation descriptors, decomposes the non-negative release rate into burst, diffusion, degradation-associated late-stage, and neural-residual components, and applies a formulation-dependent plateau through a semi-analytical state map. The model was evaluated on a literature-curated dataset containing 321 in vitro release curves, 4913 observations, 89 drugs, and 113 publications using DOI-grouped five-fold cross-validation, complementary extrapolation and sparse-sampling protocols, synthetic mechanism-recovery experiments, and retrospective orthogonal consistency analysis. **Results**: MC-NODE achieved an RMSE of 0.094±0.005 and an $R^{2}$ of 0.854±0.018, with all 321 out-of-fold trajectories satisfying monotonicity and range criteria. It recovered synthetic contribution labels more accurately than the ablated variants. The degradation-associated late-stage contribution showed positive associations with experimental degradation, molecular-weight loss, pore-evolution, and mass-loss indicators, while the diffusion contribution was positively associated with an experimental diffusion indicator. Dominant-process agreement was 83.3%, and matched external or out-of-fold trajectories achieved an RMSE of 0.108±0.020. Under drug-grouped, chemical-cluster, and alternative sparse-sampling evaluations, MC-NODE retained the lowest absolute trajectory-level errors among the compared models. **Conclusions**: MC-NODE improves formulation-level PLGA release prediction while preserving continuous, monotonic, and bounded trajectories. Its component outputs provide experimentally supported, model-attributed summaries for comparative formulation analysis.

## 1. Introduction

Poly(lactide-co-glycolide) (PLGA) microspheres are widely used for long-acting drug delivery because PLGA combines biodegradability, biocompatibility, adjustable degradation, and compatibility with diverse therapeutic compounds [1]. Drug release from PLGA microspheres arises from several coupled processes, including the rapid liberation of surface-associated drug, diffusion through the polymer matrix and aqueous pores, polymer hydrolysis, pore evolution, swelling, and erosion [2]. The relative influence of these processes may change throughout the release period, producing monophasic, biphasic, or triphasic profiles with distinct early, sustained, and late-stage behavior [3]. Similar mechanism assignments have been discussed in PLGA microsphere studies, where initial burst release has been associated with drug located near outer microsphere layers, whereas delayed release has been attributed to slower core diffusion and degradation-associated matrix changes followed by pore formation and erosion [4]. Recent PLGA–Pluronic F127 microspheres have also shown delayed and sustained release behavior, further illustrating how formulation design can shift the balance among early release, transport, and late-stage release [5]. Release kinetics also depend on drug properties, PLGA molecular weight and composition, particle size, drug loading, encapsulation characteristics, and manufacturing conditions. The interaction of these factors across multiple temporal scales makes formulation-level prediction difficult. This complexity is reflected in the literature-curated PLGA microparticle dataset of Bao et al., which contains 321 in vitro release curves, 4913 temporal observations, 89 drugs, and data collected from 113 publications [6]. Reliable prediction of complete release trajectories could reduce experimental screening requirements and support more systematic comparison of candidate formulations.

Controlled-release formulation development still relies heavily on iterative preparation and dissolution testing. Experimental studies can evaluate only a limited subset of the feasible combinations of drug, polymer, and formulation variables, which motivates computational models that connect formulation characteristics with release behavior [7]. Recent pharmaceutical work has further emphasized that PLGA-based controlled-delivery systems require coordinated optimization of polymer properties, fabrication strategy, additives, processing aids, degradation behavior, and release kinetics [8]. The interpretation of PLGA release data also depends on the in vitro release method, because sink condition, surfactant use, pH, temperature, and dissolution medium can alter the observed release profile [9]. Conventional kinetic equations, including first-order, Higuchi, Korsmeyer–Peppas, and Weibull models, provide compact descriptions of individual release curves. Their parameters are usually fitted separately for each experiment, and a single predefined equation may have limited flexibility when the dominant release process changes over time. Machine learning methods provide an alternative formulation-level approach by learning nonlinear relationships between material descriptors and release measurements. Bannigan et al. demonstrated that data-driven models can predict release from polymeric long-acting injectables and support formulation design [10]. Related work has shown that material descriptors and processing information can support predictive modelling across polymeric biomaterials [11], while recent reviews have identified machine learning as an increasingly important tool for polymeric drug-delivery development [12]. Data-driven optimization has also been applied to PLGA particle production [13], and recently released nanoparticle formulation data have expanded the available information for small-molecule delivery modelling [14]. Targeted lipid-nanoparticle studies further illustrate how carrier composition and cell-specific targeting can be coordinated to support complex therapeutic delivery objectives [15]. Comparable materials-design studies have used multi-objective prediction and inverse design to map target properties back to candidate compositions [16].

Recent studies have extended machine learning from static formulation properties to complete drug-release behavior. Literature-derived PLGA data have been combined with regression, Gaussian-process, and neural-network models and compared with newly generated in vitro measurements [17]. Song et al. recently developed an interpretable two-stage machine-learning framework for early and full release prediction in PLGA microspheres, using release-profile information to support time-dependent prediction and model interpretation [18]. Raman spectra have been used to predict drug release from functional coatings, showing that indirect measurements can contain information related to subsequent release dynamics [19]. Multi-output models have also been developed to estimate complete dissolution profiles and kinetic parameters from tablet formulations [20]. Automated active learning has further connected release prediction with sequential formulation selection in hydrogel systems [21]. Attention-based sequence-to-sequence models in other forecasting domains have also demonstrated the value of multiscale decomposition and explicit temporal context for nonstationary trajectories [22]. These studies establish the feasibility of data-driven release modelling. Many existing approaches represent time as an ordinary input variable and predict each release observation independently, leaving cumulative-state evolution implicit. Predictions queried across time may consequently form decreasing segments, exceed the feasible release range, or interpolate unstably between sparse observations.

Continuous-time scientific machine learning provides a suitable mathematical basis for addressing these limitations. Neural ordinary differential equations parameterize state derivatives with neural networks and represent system evolution continuously in time [23]. Latent ODE models extend this formulation to irregularly sampled observations and support evaluation at arbitrary query times [24]. Physics-informed learning further demonstrates that differential equations, initial conditions, and scientific constraints can restrict neural models to more plausible solution spaces [25]. Physics-informed neural models have also combined observation-driven inputs with system-level physical constraints for engineering state estimation [26]. Recent developments have increasingly combined continuous-time models with domain-specific structure. EARTH incorporated epidemiological transmission processes into a Neural ODE [27], while hybrid mechanistic modelling with deep transfer learning separated stable scientific knowledge from components that adapt to changing operating conditions [28]. Stability under irregular sampling and numerical integration has also become an explicit concern in neural differential modelling [29]. These studies suggest that an effective release model should combine formulation-dependent flexibility with a state representation that preserves the mathematical properties of cumulative release.

The reliability of mechanism-related outputs has become another important issue in scientific machine learning. Explicit dynamical models can provide interpretable components, and reliable mechanism attribution additionally requires agreement with independent physical evidence and stability across alternative parameterizations. Interpretable machine learning frameworks in alloy design have similarly linked feature-attribution analysis with experimental validation of candidate materials [30]. Recent work on explicit cellular dynamics has therefore evaluated learned interactions against external biological knowledge [31], and biophysical Neural ODE models have been examined on systems with known dynamics before application to observational data [32]. Hard-constrained learning has also shown that architecture-level constraints can impose feasibility directly [33]. In drug delivery, machine learning has been proposed for accelerating hydrogel formulation and release optimization, while limited data and weak interpretability remain important barriers [34]. A recent physics-informed framework for curcumin nanocarriers predicted diffusion-related kinetic parameters under monotonicity and non-negativity constraints, providing direct evidence that scientific structure can improve the reliability of release modelling [35]. Rigorous evaluation additionally requires realistic holdouts, competitive baselines, multiple metrics, and tests under limited observations [36].

Several connected gaps therefore remain in PLGA microsphere release prediction. Empirical kinetic equations provide useful curve descriptions but require access to the target trajectory for parameter fitting. Pointwise machine learning models treat cumulative-state evolution implicitly. Ordinary Neural ODE models provide continuous-time trajectories, while explicit state structure supplies guarantees of non-negative, monotonic, and bounded release. Soft penalties reduce physical violations in proportion to the selected weights, and residual violations can remain. Mechanism-decomposed models face an additional challenge because a flexible residual component may absorb dynamics that could be represented by structured release components. Literature-derived data also introduce publication-specific differences in experimental protocols and temporal sampling, making random formulation splitting an incomplete measure of generalization. A suitable model should therefore combine continuous-time prediction, structural validity, controlled mechanism decomposition, and evaluation across publications, drugs, and observation densities.

Motivated by these considerations, this paper proposes MC-NODE, a mechanism-decomposed and structure-preserving neural differential model for PLGA microsphere release prediction. A formulation encoder maps ten drug, polymer, and formulation descriptors to a latent context, while release time is supplied as a continuous query variable. The release intensity is decomposed into four components representing burst release, diffusion, degradation-associated late-stage release, and a controlled neural residual. A formulation-dependent plateau and a semi-analytical state equation generate continuous, monotonic, and bounded cumulative release trajectories. Mechanism-first optimization allows the structured components to explain the principal dynamics before the residual branch is introduced, while residual-flux regularization limits excessive dependence on unresolved neural dynamics. Flux-based attribution provides a conservative decomposition of the predicted release increment into four non-negative contributions.

The contributions of this study are threefold. First, MC-NODE provides a continuous-time release model with explicit guarantees of solution uniqueness, non-negativity, monotonicity, boundedness, and convergence towards a formulation-dependent plateau. Second, mechanism-first optimization, residual control, and flux-conserving attribution establish a mathematically consistent connection between trajectory prediction and model-attributed release components. Third, DOI-grouped, drug-grouped, sparse-time, and synthetic mechanism-recovery evaluations assess cross-study prediction, extrapolation to unseen drug identities, robustness to limited temporal observations, and empirical recoverability of contribution labels under controlled synthetic settings. Additional temporal-form, optimization, chemical-cluster, alternative-sampling, uncertainty, multicollinearity, feature-importance, reduced-descriptor, and formulation-matched orthogonal-consistency analyses evaluate the robustness and practical scope of the model under complementary protocols. Together, these contributions provide a mechanism-decomposed computational framework for PLGA microsphere release prediction and release-component attribution.

## 2. Results

### 2.1. Overall Prediction Performance

Table 1 reports the overall release-profile prediction performance under DOI-grouped five-fold cross-validation. The conventional kinetic equations are listed first as descriptive fitting references, followed by formulation-level prediction models. Since the descriptive kinetic equations were fitted directly to each observed release curve, they were excluded from the formulation-level ranking and statistical comparison. In contrast, KP-Param-LightGBM and Weibull-Param-LightGBM predicted kinetic parameters from formulation descriptors and generated release curves without using the target test profiles. They were therefore included as formulation-level predictors.

Among the formulation-level predictors, MC-NODE achieved the lowest MAE, RMSE, and nIAE, with values of 0.069±0.004, 0.094±0.005, and 0.061±0.004, respectively. It also achieved the highest $R^{2}$ of 0.854±0.018. The strongest structure-matched baseline was SingleRate-NODE, which achieved an RMSE of 0.100±0.005 and an nIAE of 0.067±0.004. Relative to SingleRate-NODE, MC-NODE reduced MAE by 6.8%, RMSE by 6.0%, and nIAE by 9.0%, while increasing $R^{2}$ by 0.017. The paired curve-level RMSE difference remained statistically significant after Holm correction.

The kinetic-parameter predictors provided an important same-task comparison with classical release modelling. KP-Param-LightGBM achieved an RMSE of 0.117±0.008 and an nIAE of 0.082±0.006, which were close to the time-conditioned MLP. Weibull-Param-LightGBM performed better, with an RMSE of 0.108±0.007, an nIAE of 0.074±0.005, and an $R^{2}$ of 0.817±0.027. This result confirms that predicting the parameters of a flexible kinetic equation from formulation descriptors can provide a stronger classical baseline than pointwise tree or MLP prediction. However, Weibull-Param-LightGBM remained less accurate than Penalty-Constrained MLP, SingleRate-NODE, and MC-NODE on the primary trajectory-level metrics.

The improvement over pointwise predictors remained clear. Compared with FT-Transformer, the strongest pointwise baseline, MC-NODE reduced MAE by 17.9%, RMSE by 16.8%, and nIAE by 21.8%, while increasing $R^{2}$ from 0.801 to 0.854. MC-NODE also outperformed the unconstrained Neural ODE and Penalty-Constrained MLP across MAE, RMSE, $R^{2}$, and nIAE. These comparisons show that the mechanism-decomposed positive-rate representation added accuracy beyond continuous-time modelling alone.

The $t_{50}$ results showed a different but informative pattern. SingleRate-NODE achieved the lowest $t_{50}$ error of 3.88±0.43 days, followed by Weibull-Param-LightGBM with 3.92±0.48 days and MC-NODE with 3.97±0.40 days. The competitive $t_{50}$ result of Weibull-Param-LightGBM suggests that kinetic-parameter prediction can capture the approximate release time scale. MC-NODE provided stronger overall trajectory reconstruction and maintained explicit monotonicity and range guarantees. The 0.09-day difference between MC-NODE and SingleRate-NODE corresponds to approximately 2.2 h. MC-NODE optimizes complete-trajectory reconstruction, while $t_{50}$ measures a single crossing-time endpoint. Product-specific target ranges determine the practical relevance of this difference.

Among the descriptive kinetic references, the Weibull equation achieved the lowest curve-fitting error, with an MAE of 0.051±0.006, an RMSE of 0.070±0.008, and an nIAE of 0.046±0.006. These values quantify retrospective curve fitting because each kinetic equation used the corresponding observed release profile to estimate its parameters. Formulation-level prediction is represented by Weibull-Param-LightGBM, which generates the release curve from formulation descriptors. The gap between the two settings illustrates the difference between fitting a known test curve and predicting an unseen formulation.

### 2.2. Physical Consistency and Release-Curve Reconstruction

**Figure 1:**
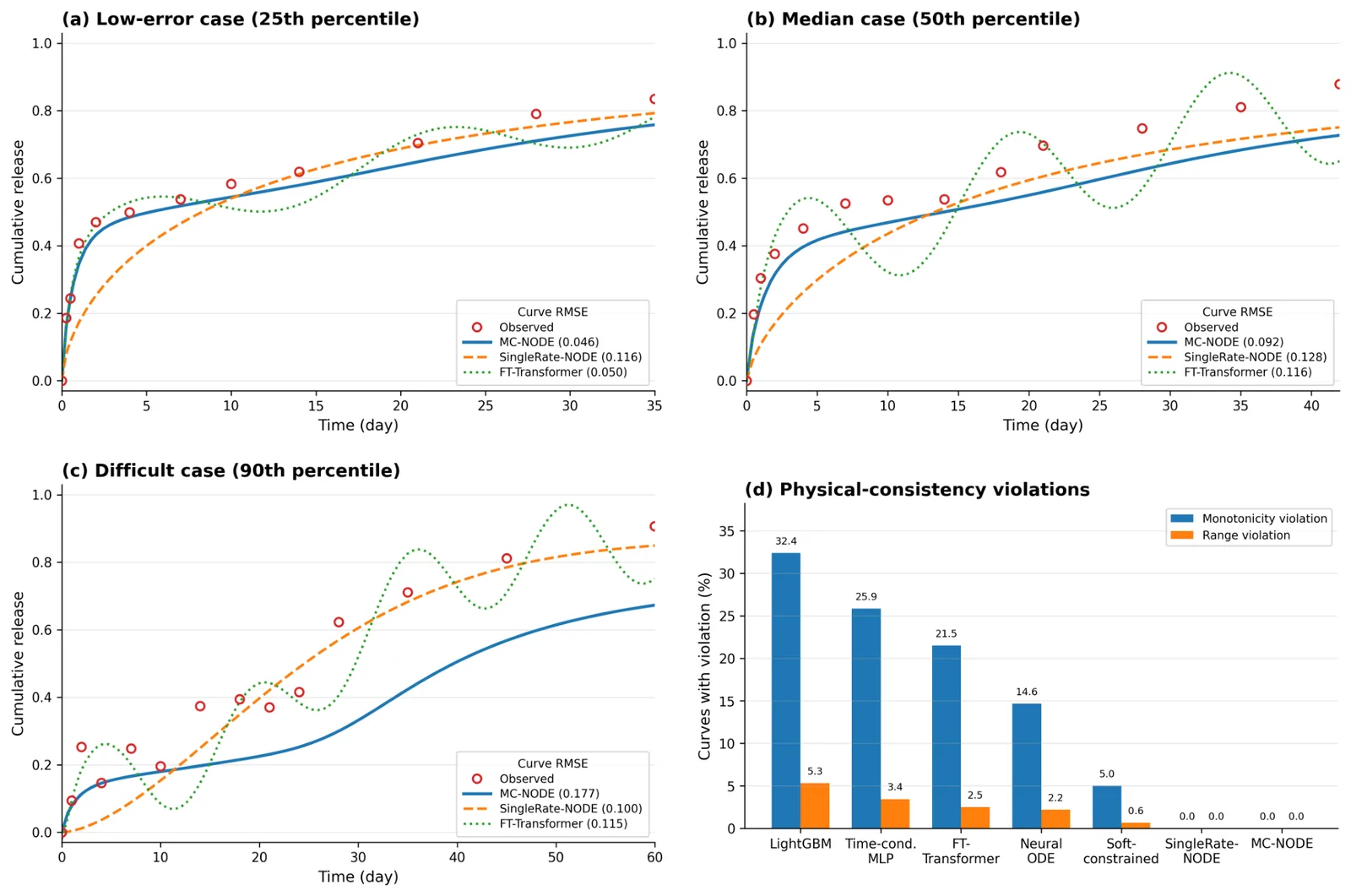
Representative release-profile reconstructions and physical-consistency evaluation. Panels (**a**–**c**) show test curves selected near the 25th, 50th, and 90th percentiles of the curve-level RMSE distribution of MC-NODE, respectively. Observed release values are shown as hollow markers, while the predicted trajectories of MC-NODE, SingleRate-NODE, and FT-Transformer are shown as continuous curves. Panel (**d**) reports the percentages of test curves containing at least one monotonicity violation or range violation. Generated using Python 3.11.9 and Matplotlib 3.8.4.

Figure 1 presents three representative release profiles and the physical-consistency results of the formulation-level predictors. The representative cases were selected from the test curves closest to the 25th, 50th, and 90th percentiles of the curve-level RMSE distribution of MC-NODE. This percentile rule selects low-error, median-error, and difficult cases objectively.

In the low-error case shown in Figure 1a, MC-NODE reconstructed both the rapid initial release and the subsequent gradual increase, obtaining a curve-level RMSE of 0.046. FT-Transformer achieved a comparable RMSE of 0.050 but produced greater local variation during the intermediate and late stages. SingleRate-NODE underestimated the initial release and obtained an RMSE of 0.116. For the median case in Figure 1b, MC-NODE achieved an RMSE of 0.092, compared with 0.128 for SingleRate-NODE and 0.116 for FT-Transformer. MC-NODE followed the overall increase of the observed profile, whereas FT-Transformer produced several local reversals and larger deviations in the middle and final portions of the release period.

The difficult case in Figure 1c contained several decreases in the observed cumulative release values, including a marked reduction between days 2 and 4. The original measurements, including these local decreases, were retained. SingleRate-NODE achieved the lowest RMSE for this individual curve at 0.100, followed by FT-Transformer at 0.115 and MC-NODE at 0.177. The monotonic trajectories of the two structure-preserving models smoothed the local decreases. MC-NODE also underestimated the later increase in release for this case. FT-Transformer followed several local changes more closely while producing a negative initial prediction and pronounced temporal oscillation.

The physical-consistency results in Figure 1d show a progressive reduction in violations as stronger temporal and structural constraints were introduced. LightGBM produced monotonicity violations in 32.4% of the test curves and range violations in 5.3%. The corresponding rates were 25.9% and 3.4% for the time-conditioned MLP, 21.5% and 2.5% for FT-Transformer, and 14.6% and 2.2% for the unconstrained Neural ODE. The Penalty-Constrained MLP reduced these values to 5.0% and 0.6%, respectively, retaining a small set of physically invalid trajectories.

Every SingleRate-NODE and MC-NODE out-of-fold trajectory satisfied the monotonicity and range criteria. This empirical result is consistent with Proposition 1, which shows that non-negative release rates and the bounded state map guarantee non-negative, monotonic, and bounded trajectories. Combined with the aggregate prediction results in Table 1, these findings show that MC-NODE maintained physically admissible release trajectories while achieving the strongest overall formulation-level prediction performance.

### 2.3. Stage-Wise Release Error Analysis

To examine whether the overall prediction advantage of MC-NODE was concentrated in a specific part of the release profile, prediction errors were further analyzed across early, middle, and late release regions. The comparison focused on four representative formulation-level predictors: FT-Transformer, Weibull-Param-LightGBM, SingleRate-NODE, and MC-NODE. These models represent attention-based pointwise prediction, classical kinetic-parameter prediction, structure-preserving single-rate dynamics, and the complete mechanism-decomposed model, respectively.

**Figure 2:**
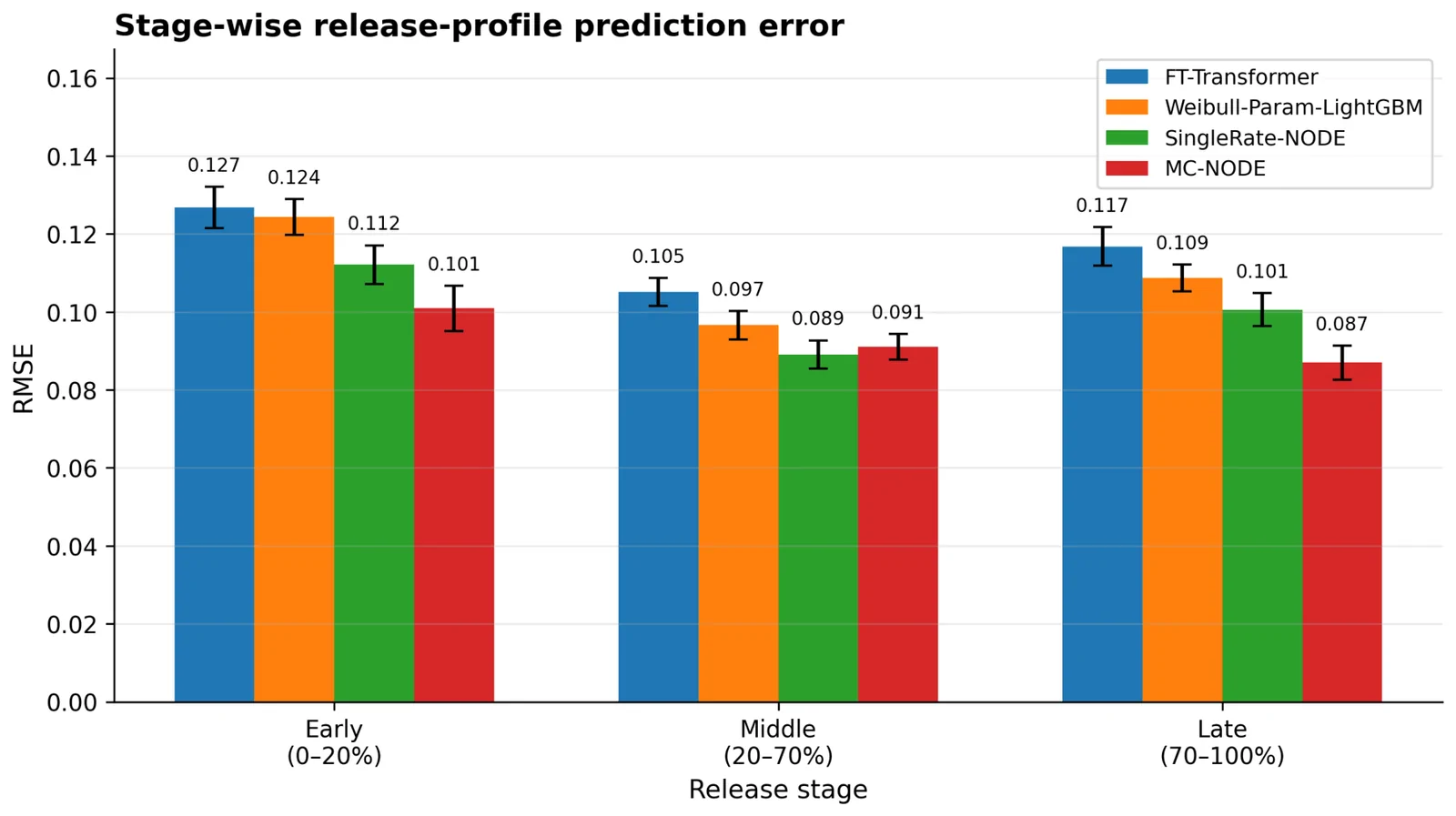
Stage-wise release-profile prediction error across early, middle, and late release regions. The time axis of each test curve was normalized and divided into early (0≤τ<0.2), middle (0.2≤τ<0.7), and late (0.7≤τ≤1.0) regions. Bars report the mean RMSE, and error bars denote the standard deviation across five random seeds after concatenating the out-of-fold predictions from the five DOI-grouped folds within each seed. Generated using Python 3.11.9 and Matplotlib 3.8.4.

As shown in Figure 2, MC-NODE achieved the lowest RMSE in the early and late release regions. In the early region, MC-NODE obtained an RMSE of 0.1009±0.0058, compared with 0.1121±0.0050 for SingleRate-NODE, 0.1244±0.0046 for Weibull-Param-LightGBM, and 0.1268±0.0053 for FT-Transformer. Relative to MC-NODE, the early-stage RMSE values of these baselines were higher by 11.1%, 23.2%, and 25.7%, respectively. This result suggests that the explicit burst branch helped capture rapid early release behavior that was more difficult for pointwise predictors and fixed-form kinetic-parameter predictors.

The middle region showed a smaller separation between the structure-preserving models. SingleRate-NODE achieved the lowest middle-stage RMSE of 0.0891±0.0036, while MC-NODE obtained a closely comparable value of 0.0910±0.0033. Weibull-Param-LightGBM achieved 0.0966±0.0037, and FT-Transformer achieved 0.1051±0.0036. This result shows that SingleRate-NODE already captures the smoother sustained-release portion of many curves, while the clearest MC-NODE gains occur during rapid early changes and terminal release.

The late region showed the clearest benefit of MC-NODE. Its late-stage RMSE was 0.0870±0.0044, compared with 0.1006±0.0042 for SingleRate-NODE, 0.1087±0.0035 for Weibull-Param-LightGBM, and 0.1168±0.0049 for FT-Transformer. These values correspond to relative increases of 15.6%, 25.0%, and 34.3% over MC-NODE. The improvement in the late region is consistent with the roles of the formulation-dependent plateau and the degradation-associated late-stage branch, which help represent terminal release levels and delayed release acceleration.

Overall, the stage-wise analysis provides a more detailed view of the trajectory-level results in Table 1. MC-NODE improved prediction most clearly in the early and late regions, where burst release, plateau behavior, and degradation-related dynamics are more likely to affect the profile shape. In the middle region, SingleRate-NODE was marginally stronger. Together, these findings localize the overall benefit of MC-NODE to phase-specific early and late release modelling.

### 2.4. Robustness to Temporal Forms and Optimization Settings

Table 2 compares alternative analytical choices for the structured branches. Replacing one temporal form yielded real-data RMSE values of 0.0937–0.0959, close to 0.0940 for the default model. The alternative diffusion form was marginally lower in RMSE, whereas the all-alternative model increased RMSE to 0.0981. Contribution recovery showed greater sensitivity: synthetic Condition C contribution MAE increased from 8.90 percentage points for the default model to 9.80–10.50 for single replacements and 12.60 when all three forms were replaced. These results demonstrate robust total-trajectory prediction under each single substitution and quantify the stronger basis dependence of component allocation.

The schedule analysis in Table 3 shows stable prediction across the evaluated stage allocations. The validation-adaptive schedule achieved a comparable RMSE of 0.0938±0.0053 and used 504±30 epochs, compared with 0.0940±0.0050 and 548±22 epochs for the default schedule. Residual activation timing primarily changed the attribution balance: early activation increased the residual contribution to 18.68±1.13%, while late activation reduced it to 10.18±4.10%. Doubling the learning rates produced the clearest deterioration, with RMSE increasing to 0.0992±0.0080.

Table 4 shows the expected trade-off for residual control. Removing residual regularization increased the residual contribution to 34.10±5.80% and synthetic contribution MAE to 13.20±1.90 percentage points. Strong regularization reduced the residual contribution to 5.76±4.01% but increased RMSE to 0.0987±0.0064. Although α=0 produced a slightly lower MAE of 0.0680±0.0050 than the default value of 0.0690±0.0040, the default $\alpha =10^{-2}$ and $\beta =10^{-5}$ provided the best overall balance across trajectory RMSE, nIAE, residual allocation, and synthetic recovery. Changes in β produced smaller effects than changes in α.

### 2.5. Chemical Extrapolation and Applicability Domain

The fingerprint-cluster holdout was more difficult than the DOI- and drug-grouped settings. As shown in Table 5, MC-NODE achieved an RMSE of 0.130±0.011, compared with 0.137±0.011 for SingleRate-NODE and 0.151±0.013 for FT-Transformer. The MC-NODE RMSE was 38.3% higher than the DOI-grouped value of 0.094 and 9.4% higher than the drug-grouped value of 0.1188, confirming that chemical separation is stricter than an unseen-identity split. MC-NODE nevertheless retained the lowest MAE, RMSE, and nIAE among the compared models.

Performance declined monotonically as chemical similarity decreased. In Table 6, MC-NODE RMSE increased from 0.110±0.009 in the high-similarity stratum to 0.149±0.012 in the low-similarity stratum. Across all 321 curves, RMSE correlated negatively with maximum test-to-training Tanimoto similarity (ρ=−0.47) and positively with standardized physicochemical distance (ρ=0.41). The same ordering was observed for the two baselines.

Drug classes were operationalized using reproducible fingerprint-similarity groups and edge-of-space physicochemical classes supported by the available molecular descriptors. The largest errors were concentrated in physicochemical regions at the edge of the observed descriptor space (Table 7). Very hydrophilic outliers showed the highest subgroup RMSE of 0.162, followed by high-molecular-weight/high-polar-surface-area formulations at 0.158 and highly lipophilic/low-polar-surface-area formulations at 0.154.

### 2.6. Alternative Sparse Sampling and Trajectory Uncertainty

Table 8 shows that retaining both endpoints made the original sparse-time protocol less difficult, while MC-NODE retained the same model ranking across all endpoint rules. At 25% retention, MC-NODE RMSE increased from 0.1160±0.0123 under Anchored sampling to 0.1285±0.0135 under unrestricted Random sampling and 0.1398±0.0150 under Interior-only sampling. Removing the final endpoint produced a larger penalty than removing the first endpoint, consistent with the importance of terminal plateau information. At 50% retention, No-first sampling increased MC-NODE RMSE by 6.6% relative to Anchored sampling. MC-NODE achieved lower RMSE and nIAE than SingleRate-NODE in every corresponding scenario.

The conformal trajectory bands in Table 9 achieved coverage close to the nominal 90% level. Under DOI-grouped validation, whole-trajectory coverage was 90.7±1.8%, and the mean band width was 0.162±0.012. Under chemical-cluster OOD, coverage was 89.6±2.4% and mean width increased to 0.218±0.018. Band width correlated with curve RMSE in both settings (ρ=0.61 and 0.66), while the highest-uncertainty quartile had substantially larger RMSE than the corresponding overall result. The intervals therefore identify extrapolations that warrant additional experimental confirmation.

### 2.7. Descriptor Diagnostics, Simplification, and Computational Cost

The descriptor matrix showed moderate dependence. The largest absolute pairwise Spearman correlation was 0.68 between the initial drug-to-polymer ratio and drug loading, followed by 0.57 between drug loading and encapsulation efficiency. All VIFs were below 5, with a maximum of 3.36 for drug loading, and the condition number of the standardized descriptor matrix was 8.6. The nonlinear formulation encoder represents descriptor interactions directly, and grouped permutation quantifies the joint influence of correlated variables.

The combined VIF and permutation-importance results are reported in Table 10. PLGA molecular weight showed the largest overall and late-stage importance, with ΔRMSE values of 0.0095±0.0018 and 0.0131±0.0022. Particle size and drug loading were most influential during early release, with early ΔRMSE values of 0.0127±0.0021 and 0.0120±0.0020. Solubility-enhancer concentration had the lowest overall importance. Grouped permutation further produced ΔRMSE values of 0.0148 for the initial-ratio/drug-loading pair and 0.0187 in the late region for the PLGA-molecular-weight/LA:GA pair. The rankings quantify predictive dependence within this dataset.

The nine-descriptor variant that omitted solubility-enhancer concentration yielded an RMSE of 0.0946±0.0053, close to the full-model value of 0.0940±0.0050, while the eight-descriptor variant that also omitted drug molecular weight increased RMSE to 0.0970±0.0058 (Table 11). The nine-descriptor variant provides a compact option when enhancer concentration is unavailable. The complete ten-descriptor model remains the primary benchmark because it preserves all consistently observed information.

The complexity comparison in Table 12 shows that mechanism decomposition increased parameter count by only 2.9% (42,912 to 44,176 parameters). Under the common benchmark, training time per fold increased from 4.80±0.25 to 6.30±0.32 min, latency from 0.84±0.05 to 1.21±0.07 ms per formulation, and peak GPU memory from 0.42±0.02 to 0.48±0.02 GB. These increases correspond to approximately 1.5 min per fold, 0.37 ms per formulation, and 0.06 GB, respectively.

### 2.8. Orthogonal Consistency with Experimental Mechanism Indicators

The retrospective formulation-matched analysis showed convergent positive associations between MC-NODE attribution and independent experimental measurements (Table 13). The degradation-associated late-stage contribution correlated with the measured degradation rate (Spearman ρ=0.555, N=13), polymer molecular-weight loss (ρ=0.527, N=13), pore evolution (ρ=0.738, N=8), and polymer mass loss (ρ=0.476, N=8). The strongest association was observed for pore evolution.

The diffusion contribution was positively associated with the experimental diffusion indicator (ρ=0.599, N=8). Across the 15 formulations with paired temporal indicators, the predicted late-stage onset had an MAE of 4.95 days and a median absolute error of 4.20 days. The predicted dominant structured process agreed with the experimental process assignment in 15 of 18 formulations, corresponding to 83.3% agreement. The external or out-of-fold release trajectories for the 18 matched formulations achieved a mean RMSE of 0.108±0.020.

These results provide formulation-level orthogonal consistency across complementary degradation-, structure-, and transport-related measurements. Together, the indicators provide direct experimental support for the model-attributed component summaries.

## 3. Discussion

### 3.1. Ablation Study

Table 14 evaluates the contribution of the major components of MC-NODE under DOI-grouped five-fold cross-validation. The complete model achieved the lowest RMSE and nIAE and the highest $R^{2}$, indicating that the proposed components jointly improved the reconstruction of complete release trajectories. Removing mechanism decomposition increased RMSE from 0.094 to 0.100 and nIAE from 0.061 to 0.067. This degradation shows that the structured burst, diffusion, and degradation-associated late-stage branches provided additional predictive value beyond the positive-rate analytical architecture represented by SingleRate-NODE.

Removing the neural-residual branch increased RMSE to 0.099 and reduced $R^{2}$ to 0.840. The structured components captured the principal release patterns, and the residual branch added flexibility for formulation-specific dynamics beyond the predefined temporal components. The moderate performance change after residual removal shows that the structured branches account for most of the predictive signal.

The mechanism-first optimization strategy substantially affected the allocation of release dynamics among the model components. Activating all branches from the beginning of training increased the mean residual contribution from 12.6% to 24.8% and increased RMSE from 0.094 to 0.098. Removing residual control produced a larger residual contribution of 34.1%. This variant obtained a slightly lower MAE of 0.068, together with higher RMSE and nIAE and lower $R^{2}$ than the complete model. Its higher RMSE and residual-contribution variability further indicates less stable decomposition. Mechanism-first optimization and residual regularization consequently concentrate the allocation in the structured branches while preserving residual flexibility.

Fixing the final release plateau to one caused the largest overall deterioration. RMSE increased to 0.103, nIAE increased to 0.070, and $R^{2}$ decreased to 0.829. This result shows that a shared unit plateau produces systematic error across formulations with incomplete release. The formulation-dependent plateau head allowed MC-NODE to represent differences in terminal release levels and reduced systematic errors near the later part of the predicted trajectories.

Overall, the ablation results show complementary roles for the principal components of MC-NODE. Mechanism decomposition improved trajectory prediction relative to a single positive-rate function, the neural residual represented unresolved formulation-dependent variation, and the mechanism-first strategy controlled how much of the release process was assigned to that flexible component. The learned plateau produced the largest individual improvement, while residual control provided a small trade-off in pointwise MAE in exchange for lower trajectory-level error and a substantially more restrained mechanism decomposition.

### 3.2. Terminal-Release-Stratified Plateau Analysis

The ablation study showed that replacing the formulation-dependent plateau with a fixed value of one caused the largest overall degradation among the examined MC-NODE variants. To further analyze this effect, we stratified the 321 test curves according to their observed final cumulative release fraction and report the low, medium, and high terminal-release groups in Table 15. This analysis was designed to determine whether the learned plateau head primarily improved prediction for incomplete-release profiles, where assuming complete release would be expected to introduce systematic late-stage error.

The three terminal-release groups contained 82, 134, and 105 curves, respectively, with mean final release values of 0.683±0.041, 0.823±0.043, and 0.948±0.030. This stratification provides a direct test of the plateau head because the fixed-complete-release assumption is expected to be most problematic in the low and medium terminal-release groups and least problematic when the observed final release is close to one.

In the low terminal-release group, MC-NODE achieved the strongest performance across all reported metrics. Its full-profile RMSE was 0.102±0.006, compared with 0.111±0.006 for SingleRate-NODE, 0.121±0.006 for Weibull-Param-LightGBM, and 0.128±0.007 for FT-Transformer. The advantage was more pronounced for late-stage and final-release metrics. MC-NODE reduced late RMSE to 0.091±0.006 and final-release MAE to 0.055±0.007, whereas the Fixed Plateau ablation produced a late RMSE of 0.145±0.009 and a final-release MAE of 0.122±0.013. The Fixed Plateau variant also showed a strong positive final-release bias of +0.104±0.015, indicating systematic overprediction when incomplete-release curves were forced towards a plateau of one. In contrast, MC-NODE reduced this bias to +0.008±0.008.

A similar pattern was observed in the medium terminal-release group. MC-NODE obtained the lowest full-profile RMSE (0.091±0.004), nIAE (0.059±0.004), late RMSE (0.086±0.004), and final-release MAE (0.044±0.006). SingleRate-NODE was the strongest non-ablated baseline, but its final-release MAE remained higher at 0.062±0.006. The Fixed Plateau ablation again overestimated final release, with a bias of +0.061±0.010, while MC-NODE showed a near-zero bias of −0.003±0.006. These results indicate that the learned plateau head was particularly important for formulations whose terminal release was substantially below complete release.

The high terminal-release group showed a different but informative pattern. Because the observed final release was close to one (0.948±0.030), the fixed-complete-release assumption was less harmful. Fixed Plateau achieved the lowest full-profile RMSE (0.087±0.005), nIAE (0.057±0.004), and final-release MAE (0.039±0.005), while MC-NODE achieved a very similar full-profile RMSE of 0.089±0.005 and the lowest late-stage RMSE of 0.084±0.005. This result supports the interpretation that a fixed plateau can be adequate when formulations approach complete release, but becomes inappropriate for low and medium terminal-release profiles.

Overall, the terminal-release-stratified analysis explains why the formulation-dependent plateau was the most important individual component in the ablation study. The plateau head reduced systematic terminal-release overprediction in curves with incomplete release and improved late-stage reconstruction. The benefit was largest in the low and medium terminal-release groups, where assuming complete release produced substantial positive bias. In contrast, the gap between Fixed Plateau and MC-NODE narrowed in the high terminal-release group, consistent with the fact that those curves already approached complete release. These findings support the use of a formulation-dependent plateau for literature-derived PLGA release data, where terminal release levels vary substantially across formulations. The 82 curves in the 0.60–0.75 group quantify behavior close to the source-dataset inclusion boundary. The reported applicability domain covers terminal cumulative release values of 0.60 or higher.

### 3.3. Synthetic Mechanism Recovery

Component-level ground-truth labels were available in the controlled synthetic setting. The compact label “erosion” is retained in the synthetic class names and plotted figure labels. The synthetic benchmark therefore evaluates recovery of known generating contributions. Three conditions were considered. Condition A used a matched generator without observation noise, Condition B used the same generator with 50% temporal observation retention and Gaussian observation noise, and Condition C used a mildly mismatched generator with the same sparse and noisy observation setting. Each condition contained balanced burst-dominant, diffusion-dominant, erosion-dominant, and mixed scenarios. The test split contained 180 curves for each condition.

Under the ideal matched condition, MC-NODE achieved a curve RMSE of 0.011±0.002, a contribution MAE of 2.80±0.50 percentage points, a dominant-class accuracy of 96.3±1.7%, and a mean Spearman correlation of 0.956±0.018. Removing the neural-residual branch increased contribution MAE to 6.40±0.90 percentage points and reduced dominant-class accuracy to 88.6±2.8%. Removing mechanism-first training also degraded contribution recovery, with a contribution MAE of 5.10±0.80 percentage points and an accuracy of 91.7±2.4%. The variant without residual control obtained a slightly lower curve RMSE of 0.010±0.002, but its contribution MAE increased to 7.20±1.10 percentage points and its dominant-class accuracy decreased to 86.9±3.4%. This result shows that comparable curve error can coexist with weaker contribution recovery.

The sparse and noisy matched condition increased the difficulty of mechanism recovery. MC-NODE maintained the lowest contribution MAE of 5.90±0.90 percentage points and the highest dominant-class accuracy of 90.2±2.5%. The corresponding curve RMSE was 0.034±0.005, and the mean Spearman correlation was 0.884±0.030. The ablation variants showed larger attribution errors. In particular, removing the residual branch increased contribution MAE to 10.10±1.40 percentage points, while removing residual control increased it to 11.60±1.80 percentage points. The latter variant again achieved a comparable curve RMSE of 0.033±0.006 but produced the lowest dominant-class accuracy of 76.1±4.6% among the compared models. These results show that residual control primarily improves decomposition reliability.

**Figure 3:**
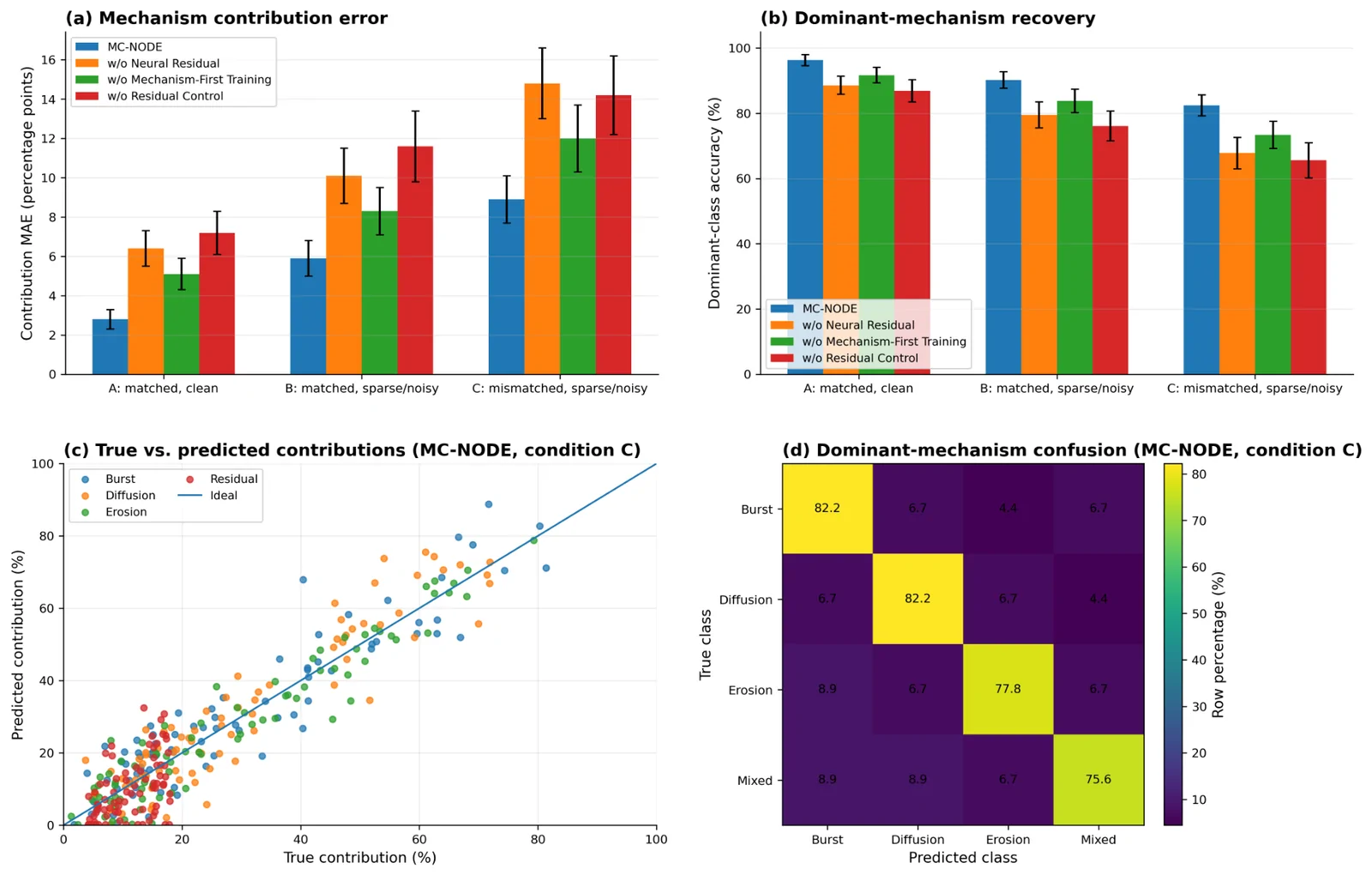
Synthetic mechanism-recovery evaluation. Panel (**a**) reports the contribution MAE in percentage points under three synthetic conditions. Panel (**b**) reports dominant-mechanism classification accuracy. Panel (**c**) shows true and predicted mechanism contributions for MC-NODE under the mismatched sparse/noisy condition. Panel (**d**) shows the row-normalized dominant-mechanism confusion matrix for MC-NODE under the same condition. The label “erosion” denotes the degradation-associated late-stage component. Error bars in panels (**a**,**b**) denote the standard deviation across five random seeds. Generated using Python 3.11.9 and Matplotlib 3.8.4.

Under the mismatched sparse/noisy condition, all models showed lower mechanism-recovery performance, as expected. MC-NODE still achieved the best overall recovery, with a curve RMSE of 0.049±0.007, a contribution MAE of 8.90±1.20 percentage points, a dominant-class accuracy of 82.4±3.2%, and a mean Spearman correlation of 0.781±0.045. The variant without the neural-residual branch obtained a contribution MAE of 14.80±1.80 percentage points, showing the value of a residual pathway under structural mismatch. Removing mechanism-first training and residual control also degraded mechanism recovery, with contribution MAE values of 12.00±1.70 and 14.20±2.00 percentage points, respectively. The scatter plot in Figure 3c shows that MC-NODE retained a positive association between true and predicted contributions even under structural mismatch, while the confusion matrix in Figure 3d remained dominated by its diagonal entries.

Overall, the synthetic benchmark supports the empirical recoverability of the mechanism-related components. The complete MC-NODE model consistently achieved the lowest contribution error and the highest dominant-class accuracy across the three conditions. The ablation results further show that the neural-residual branch, mechanism-first optimization, and residual control have complementary roles. The residual branch improves representation under heterogeneous or mismatched dynamics, whereas mechanism-first optimization and residual control prevent the flexible component from absorbing dynamics that can be assigned to the structured release branches.

### 3.4. Mechanism Attribution and Case Analysis

**Figure 4:**
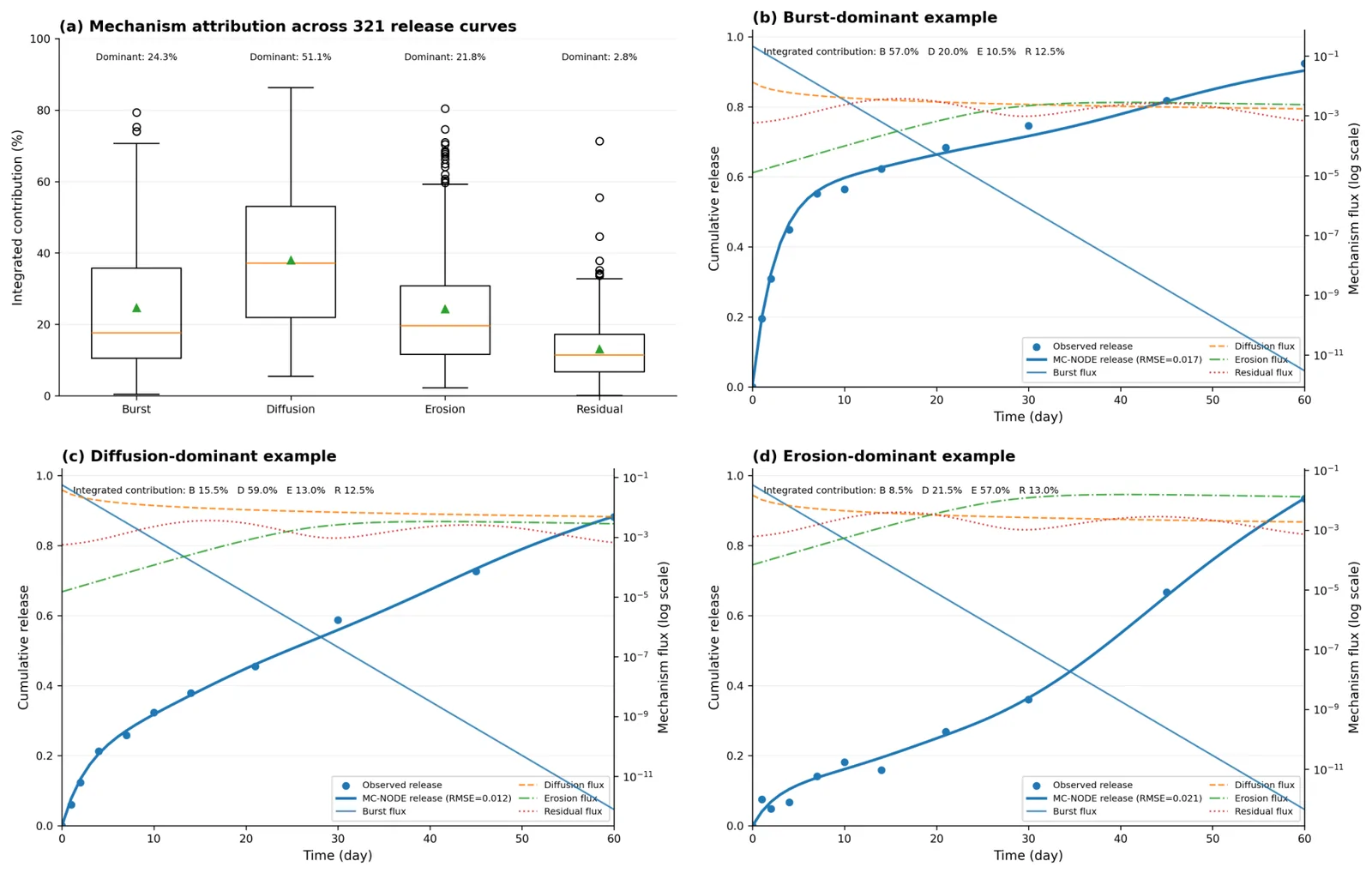
Mechanism attribution and representative release cases. Panel (**a**) shows the distributions of the integrated burst, diffusion, erosion, and residual contributions across 321 out-of-fold release curves. In the box plots, the boxes represent the interquartile range, the orange horizontal lines indicate the medians, the green triangles indicate the means, and the whiskers extend to the most extreme observations within 1.5×IQR of the lower and upper quartiles. Open circles denote observations outside the whisker range. The plotted label “erosion” denotes the degradation-associated late-stage component. The annotations report the proportion of curves for which each component was dominant. Panels (**b**–**d**) present burst-dominant, diffusion-dominant, and erosion-dominant examples, respectively. Observed and predicted cumulative release values are plotted against the left axis, while the mechanism-specific release fluxes are plotted on the logarithmic right axis. Generated using Python 3.11.9 and Matplotlib 3.8.4.

Figure 4 summarizes the mechanism contributions assigned by MC-NODE to the 321 out-of-fold release curves. Diffusion had the largest mean contribution at 38.0%, followed by burst release at 24.7%, the degradation-associated late-stage component at 24.3%, and the neural residual at 13.0%. Diffusion was the dominant component in 164 curves (51.1%), whereas burst release and the degradation-associated late-stage component dominated 78 (24.3%) and 70 (21.8%) curves, respectively. The residual branch was dominant in only nine curves (2.8%).

The four reported contributions form a conservative decomposition of the predicted release increment according to Proposition 2. They are reported as model-attributed release-component summaries and quantify how MC-NODE allocates the predicted release increment among burst release, diffusion, the degradation-associated late-stage component, and the neural residual.

The burst-dominant example in Figure 4b assigned 57.0% of the integrated release to the burst component, compared with 20.0% to diffusion, 10.5% to the degradation-associated late-stage component, and 12.5% to the residual. The burst flux reached its maximum at the initial time and declined rapidly, while the predicted cumulative release increased to approximately 0.56 by day 7. MC-NODE reconstructed this profile with an RMSE of 0.017, including the rapid early increase and the slower subsequent release.

The diffusion-dominant example assigned 59.0% of the released amount to diffusion, with contributions of 15.5%, 13.0%, and 12.5% from burst release, the degradation-associated late-stage component, and the residual, respectively. The diffusion flux remained active throughout the 60-day interval, producing a gradual trajectory that reached 0.88 at the final observation. The predicted curve closely followed the measured profile, with an RMSE of 0.012. This case illustrates sustained release represented primarily by the diffusion branch, with a residual contribution of 12.5%.

The late-stage-dominant example, labelled “erosion-dominant” in Figure 4d to match the compact mathematical label, exhibited a limited early increase followed by faster release later in the profile. The degradation-associated late-stage component accounted for 57.0% of the integrated contribution, whereas burst release, diffusion, and the residual accounted for 8.5%, 21.5%, and 13.0%, respectively. Its flux increased progressively and reached its maximum near day 41. Correspondingly, cumulative release increased from approximately 0.36 at day 30 to 0.67 at day 45 and 0.93 at day 60. The curve-level RMSE was 0.021.

Across the three examples, the temporal locations of the attributed components were consistent with their intended modelling roles: burst release was concentrated near the beginning, diffusion persisted over the observation period, and the degradation-associated late-stage component increased during the later stage. These patterns are consistent with commonly reported multiphase release behavior of PLGA systems [2,3]. The residual contribution remained between 12.5% and 13.0% in the selected cases and averaged 13.0% across the complete dataset, indicating that the structured components explained most of the predicted release dynamics.

The formulation-matched orthogonal analysis further linked these model-attributed components to independent measurements reserved for evaluation. Positive associations across degradation rate, molecular-weight loss, pore evolution, mass loss, and a diffusion-related indicator, together with 83.3% dominant-process agreement and a matched-trajectory RMSE of 0.108±0.020, support the intended process alignment of the structured branches. Variation in association strength is expected because the experimental indicators capture complementary aspects of the coupled release process.

### 3.5. Generalization and Robustness

**Figure 5:**
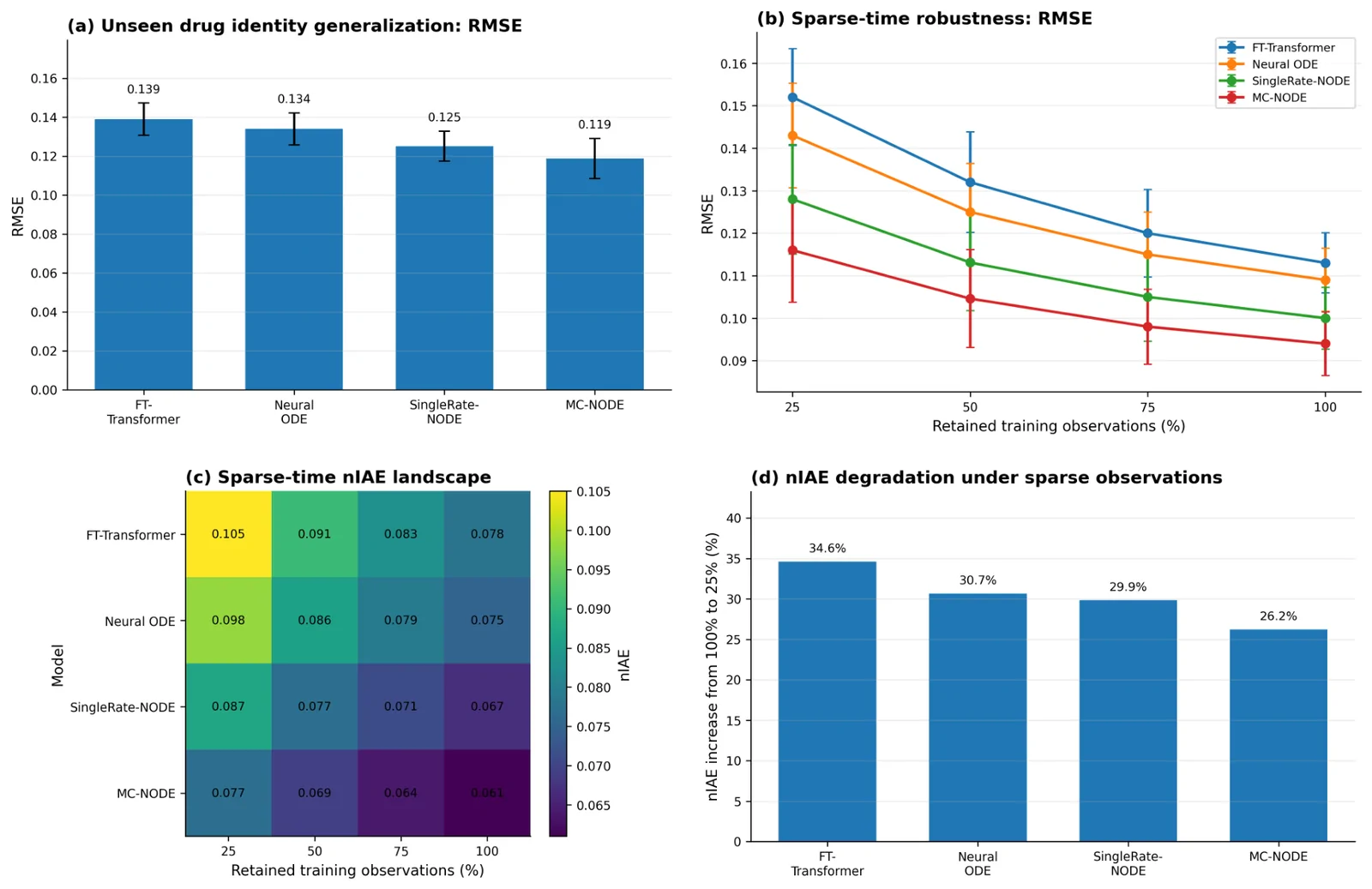
Generalization and robustness of the formulation-level predictors. Panel (**a**) reports RMSE under drug-grouped five-fold cross-validation, which evaluates prediction for unseen drug identities. Panel (**b**) shows test RMSE under sparse-time training, where 25%, 50%, 75%, or 100% of the observations in each training curve were retained and the test curves were kept complete. Panel (**c**) reports the corresponding sparse-time nIAE values as a heatmap. Panel (**d**) shows the relative nIAE increase from 100% to 25% retained training observations. Error bars in panels (**a**,**b**) denote the standard deviation across five random seeds after concatenating the out-of-fold predictions from the five folds within each seed. Generated using Python 3.11.9 and Matplotlib 3.8.4.

Figure 5 evaluates MC-NODE under two settings that differ from the main DOI-grouped prediction task. Figure 5a reports RMSE under drug-grouped five-fold cross-validation, in which all formulations associated with the same drug identity were assigned to one fold. Figure 5b reports the effect of sparse temporal supervision on RMSE when only 25%, 50%, 75%, or 100% of the observations in each training curve were retained, while every test curve remained complete. Figure 5c,d reorganize the nIAE results to avoid duplicating the RMSE trend: Figure 5c presents the absolute nIAE values as a model–retention heatmap, and Figure 5d reports the relative nIAE increase when the retained training observations were reduced from 100% to 25%.

Under the unseen-drug-identity setting, MC-NODE achieved the lowest RMSE of 0.1188±0.0103 and the lowest nIAE of 0.0785±0.0067. SingleRate-NODE was the strongest baseline, with an RMSE of 0.1252±0.0077 and an nIAE of 0.0841±0.0048. MC-NODE therefore reduced these two errors by 5.1% and 6.7%, respectively. Compared with FT-Transformer, the corresponding reductions were 14.5% and 18.2%. MC-NODE also achieved the lowest MAE of 0.0875±0.0068 and the highest $R^{2}$ of 0.7762±0.0242. SingleRate-NODE retained a small advantage for $t_{50}$ estimation, with an error of 5.255±0.245 days compared with 5.381±0.246 days for MC-NODE.

All methods showed higher errors under drug-level holdout than under the main DOI-grouped experiment, confirming that prediction for unseen drug identities was more difficult than prediction across unseen publications. The RMSE of MC-NODE increased from 0.094 to 0.1188, corresponding to a relative increase of 26.4%. MC-NODE retained the lowest absolute error across the reported trajectory-level metrics, and the increase quantifies the remaining difficulty of drug-level extrapolation. The 26.4% increase indicates that molecular weight, topological polar surface area, and LogP leave drug-specific variability unresolved, including solid-state properties, ionization, intrinsic solubility, and drug–polymer interactions. Prospective use with a novel drug should combine richer molecular representations, applicability-domain screening, and independent or small-sample calibration data.

The sparse-time experiment produced a consistent ranking at every observation-retention level. With only 25% of the training observations retained, MC-NODE achieved an RMSE of 0.1160±0.0123 and an nIAE of 0.0770±0.0076. The corresponding values were 0.1280±0.0129 and 0.0870±0.0083 for SingleRate-NODE, 0.1430±0.0123 and 0.0980±0.0078 for Neural ODE, and 0.1520±0.0114 and 0.1050±0.0073 for FT-Transformer. As the retained observations increased to 50%, 75%, and 100%, the RMSE of MC-NODE decreased to 0.1046, 0.0980, and 0.0940, respectively, while its nIAE decreased to 0.0693, 0.0640, and 0.0610.

The nIAE heatmap in Figure 5c confirms that the trajectory-level integral error followed the same model ranking as RMSE while preserving the complete numerical pattern across observation-retention levels. At full observation density, MC-NODE achieved the lowest nIAE of 0.061, followed by SingleRate-NODE, Neural ODE, and FT-Transformer. At 25% observation retention, the same ordering was maintained, with nIAE values of 0.077, 0.087, 0.098, and 0.105, respectively. The matching RMSE and nIAE rankings demonstrate lower local and integrated trajectory error for MC-NODE.

Figure 5d further summarizes the effect of temporal sparsity using the relative nIAE increase from 100% to 25% retained observations. MC-NODE showed the smallest nIAE degradation at 26.2%, compared with 29.9% for SingleRate-NODE, 30.7% for Neural ODE, and 34.6% for FT-Transformer. The corresponding RMSE increases were 23.4%, 28.0%, 31.2%, and 34.5%, respectively. The advantage of MC-NODE over SingleRate-NODE also increased as the training curves became sparser: the RMSE reduction increased from 6.0% at full observation density to 9.4% at 25% retention. These results suggest that the structured release components provided a useful temporal prior when fewer observations were available, while the residual branch retained sufficient flexibility to model formulation-specific deviations.

### 3.6. Practical Interpretation and Prospective Use

MC-NODE reduced overall RMSE by 6.0% and nIAE by 9.0% relative to the structure-matched SingleRate-NODE, with the clearest gains in early and late release. The mechanism-decomposed model used 2.9% more parameters, added approximately 0.37 ms of latency per formulation, and increased training time by about 31% under the common benchmark. SingleRate-NODE retained small advantages in middle-stage RMSE and $t_{50}$ and provides a compact option for a single release-time endpoint or a smooth sustained-release segment. MC-NODE provides the broader output needed when complete-trajectory shape, terminal behavior, uncertainty, and model-attributed component summaries are central to screening.

A prospective workflow begins after candidate microspheres have been prepared and particle size, drug loading, and encapsulation efficiency are available. MC-NODE first checks whether the drug lies within the chemical applicability domain, predicts the complete in vitro trajectory and terminal plateau, and returns a calibrated trajectory band. Candidates can then be ranked against a target profile, while low-similarity or high-uncertainty candidates are prioritized for confirmatory testing. Feature-importance results can guide which formulation variables require the tightest measurement and control. A smaller subset then proceeds to complete long-duration release testing, and the resulting measurements can support subsequent model updates.

The nine-descriptor variant omitting solubility-enhancer concentration retained nearly the same average performance and provides a compact configuration when that field is unavailable. The ten-descriptor model remains the primary configuration and preserves the complete set of consistently observed inputs.

### 3.7. Limitations and Scope of Applicability

The evaluated dataset covers emulsion-prepared PLGA microparticles with release durations of at least 72 h and terminal cumulative release of 0.60 or higher. All 321 curves met the inherited 0.60 criterion, and 82 curves fall between 0.60 and 0.75, providing evidence near the lower boundary. The current applicability domain begins at a terminal cumulative release of 0.60.

The 113 source publications used different assay protocols, sampling schedules, and reporting procedures. DOI-grouped cross-validation separates publications between training and test folds, while protocol variation remains part of the evaluation. Publication identity serves as grouping metadata because each test DOI is unseen. A protocol-aware extension can incorporate medium composition, pH, temperature, surfactant use, dissolution apparatus, and sampling procedures as cross-study coverage improves.

The ten-descriptor representation covers consistently reported drug, polymer, and formulation factors. The common representation uses the fields with consistent cross-study coverage; broader reporting of polymer end-group chemistry, crystallinity, particle morphology and porosity, residual solvent, detailed process histories, and release-protocol variables would support an expanded input space. Fingerprint-cluster validation increased MC-NODE RMSE from 0.094 to 0.130, and the low-similarity stratum reached 0.149, demonstrating reduced accuracy in chemically remote regions. The calibrated bands widened under this OOD setting and identify difficult extrapolations. Setting-specific calibration supports transfer to new manufacturing processes and assay environments.

The branch contributions are model-attributed release-component summaries. Proposition 2 establishes non-negative, conservative accounting of the predicted release increment, while overlapping temporal bases permit multiple component allocations to produce similar cumulative curves. Single temporal-form substitutions retained 90.9–92.8% dominant-component agreement with the default model, whereas replacing all three forms reduced agreement to 84.0% despite a modest RMSE increase from 0.0940 to 0.0981. The formulation-matched orthogonal analysis added direct experimental consistency across degradation-, pore-, mass-loss-, and diffusion-related indicators, with indicator-specific sample sizes of 8–13, an onset analysis of 15 formulations, and dominant-process evaluation across 18 formulations. These complementary measurements support process alignment, while larger synchronized datasets would enable finer separation of coupled degradation, erosion, morphology, and transport effects.

Permutation importance quantifies predictive dependence within the observed data, and external validation assesses the transferability of descriptor-reduced configurations. MC-NODE supports post-characterization candidate prioritization and directs selected formulations to confirmatory release testing. Controlled prospective studies can extend validation to new drug classes, manufacturing methods, release environments, and other polymeric delivery systems.

## 4. Materials and Methods

### 4.1. Dataset and Feature Processing

The experiments used the literature-curated PLGA microparticle dataset reported by Bao et al. [6]. The dataset contains 321 independent in vitro release curves, 4913 temporal observations, 89 small-molecule drugs, and 113 source publications. The source study retained emulsion-prepared PLGA microparticles with release durations of at least 72 h and final cumulative release above 60%. This threshold was inherited from the source dataset, so the evaluated terminal-release domain begins at 0.60. The dataset contains an average of 15.3 observations per curve and covers diverse drug, polymer, and formulation conditions.

Ten numerical formulation predictors were used. Drug descriptors included molecular weight, topological polar surface area, and LogP. Polymer descriptors included PLGA molecular weight and the lactide-to-glycolide ratio. Formulation descriptors included the initial drug-to-polymer ratio, particle size, drug-loading capacity, encapsulation efficiency, and solubility-enhancer concentration. Drug molecular descriptors were calculated from the reported SMILES representations using RDKit during dataset construction [6]. These fields were selected because they were defined consistently across the curated records and jointly represent drug, polymer, and formulation factors. The common input representation therefore focused on these ten fields; polymer end-group chemistry, crystallinity, particle morphology and porosity, residual solvent, detailed manufacturing conditions, and release-protocol variables showed sparse or heterogeneous cross-study coverage. Release time in days was treated as a continuous query variable, and cumulative drug release was represented as a fraction between zero and one.

A curve-level quality-control procedure was applied before constructing the experimental partitions. Each retained curve was required to contain at least six distinct observation times, include at least one measurement within the first 24 h, extend for at least 72 h, and reach a final cumulative release of at least 0.60. Observation times were required to be non-negative, and all ten formulation predictors were required to be available. Drug and polymer molecular weights, particle size, and the initial drug-to-polymer ratio were required to be positive, while drug-loading capacity, encapsulation efficiency, and solubility-enhancer concentration were restricted to the interval from 0 to 100%. All 321 release curves satisfied these criteria and were retained for modelling. All 321 source curves met the repeated terminal-release check and remained in the modelling set.

Records with the same formulation identifier and observation time were merged by averaging their release values. Local decreases between consecutive observations were retained because they may reflect experimental variability or curve digitization noise. The measured targets retained their original local decreases. Records were grouped by formulation identifier and ordered by time to reconstruct complete release trajectories. Drug identity and publication DOI were retained as grouping metadata and were excluded from the predictive input.

Continuous formulation predictors were standardized using the mean and standard deviation estimated from the training partition of each experimental split. The same transformation was subsequently applied to the corresponding validation and test partitions. Release time retained its original unit of days. The structured mechanism branches received the original time *t*, while the neural-residual branch received both *t* and log(1+t) to increase temporal resolution during early release. The cumulative release target remained on its original fractional scale.

### 4.2. MC-NODE Model

For the *i*th PLGA microsphere formulation, let $x_{i}\in R^{10}$ denote the formulation vector and let ${\left\{\left(t_{ij},R_{ij}\right)\right\}}_{j=1}^{n_{i}}$ denote the observed release profile. The prediction task is formulated as

(1) $$D_{i}={\left\{\left(t_{ij},R_{ij}\right)\right\}}_{j=1}^{n_{i}},\;{\hat{R}}_{i}\left(t\right)=f_{\Theta }\left(x_{i},t\right),$$

where $t_{ij}\geq 0$ is the *j*th observation time, $R_{ij}\in \left[0,1\right]$ is the measured cumulative release fraction, $n_{i}$ is the number of observations for formulation *i*, and Θ denotes the trainable model parameters. All observations belonging to the same formulation share one formulation context and one set of release parameters, while the query time determines the position on the predicted trajectory.

**Figure 6:**
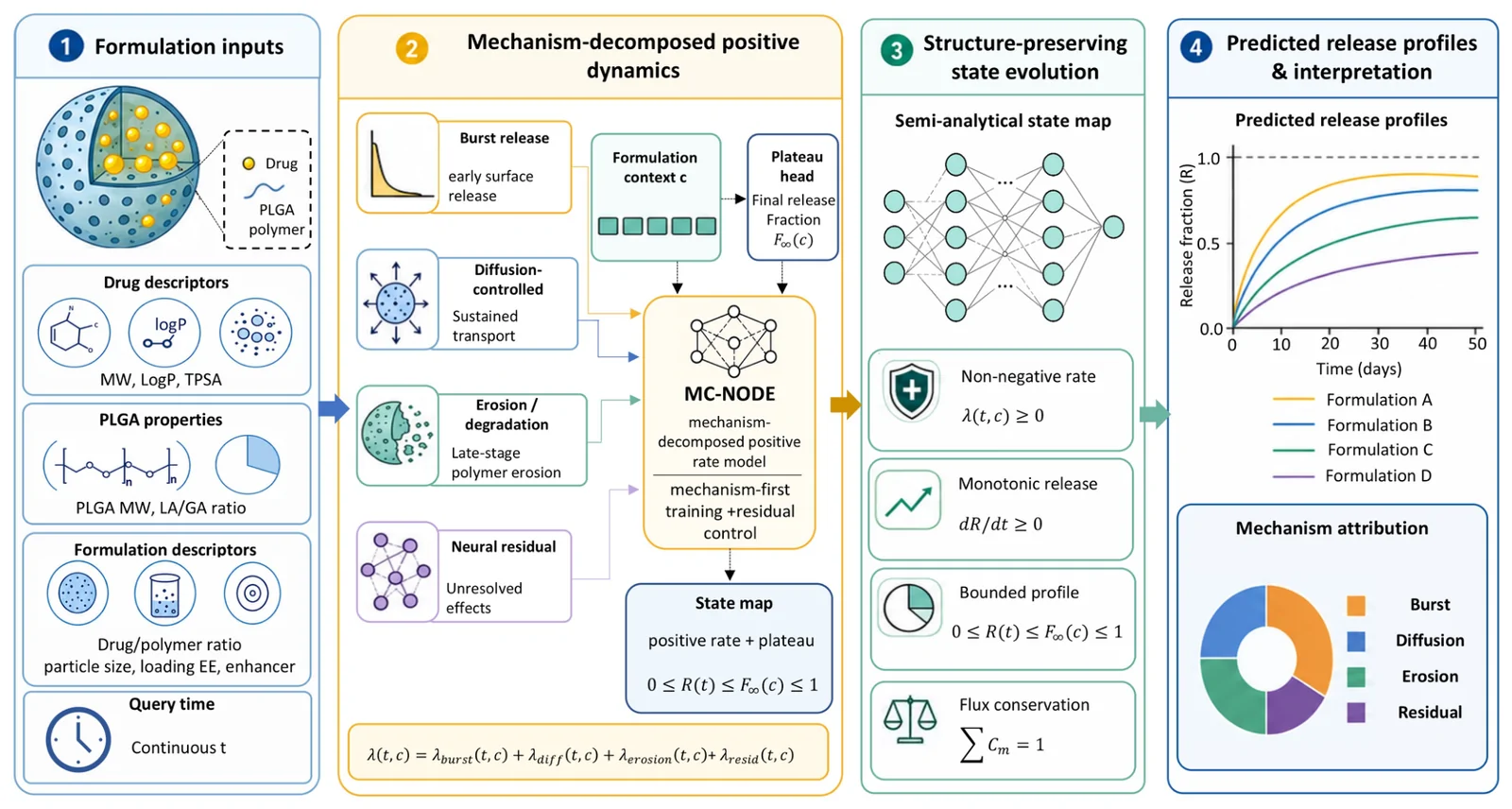
Architecture of MC-NODE. Ten drug, polymer, and formulation descriptors are encoded into a formulation context, while release time is supplied as a continuous query variable. Burst, diffusion, degradation-associated late-stage, and neural-residual branches construct a mechanism-decomposed non-negative release rate. A formulation-dependent plateau head estimates the final releasable fraction, and a semi-analytical structure-preserving state map generates a continuous, monotonic, and bounded cumulative release trajectory. Mechanism-first optimization and residual control restrict excessive dependence on the neural residual, while flux-based attribution decomposes the predicted release increment into burst, diffusion, late-stage, and residual contributions. Created using Microsoft PowerPoint for Microsoft 365, Version 2506; curves were generated using Python 3.11.9 and Matplotlib 3.8.4. Selected non-data icons were generated with ChatGPT-5.5 image generation (OpenAI) and edited by the authors.

Figure 6 provides an overview of the MC-NODE architecture. The formulation descriptors are encoded into a latent context, and a separate plateau head estimates the formulation-dependent final releasable fraction. The burst, diffusion, degradation-associated late-stage, and neural-residual branches construct a non-negative release-rate function. This rate is then converted into a continuous cumulative release profile through a semi-analytical structure-preserving state map, and the same forward computation provides flux-based release-component attribution.

A compact MLP encoder maps the formulation vector to a latent context. A separate plateau head estimates the formulation-dependent upper limit of cumulative release:

(2) $$c_{i}=E_{\phi }\left(x_{i}\right),\;F_{\infty }\left(c_{i}\right)=\sigma \left(g_{\psi }\left(c_{i}\right)\right),$$

where $E_{\phi }$ denotes the formulation encoder, $g_{\psi }$ denotes the plateau head, and σ(·) is the sigmoid function. The predicted plateau satisfies $0<F_{\infty }\left(c_{i}\right)<1$. This separation allows the model to distinguish release capacity from release speed, since two formulations may exhibit similar early release rates while approaching different terminal fractions.

The total release rate is decomposed into four non-negative components:

(3) $$\begin{array}{cc} \lambda (t,c)\; & =\lambda_{\mathrm{burst}}\left(t,c\right)+\lambda_{\mathrm{diff}}\left(t,c\right)+\lambda_{\mathrm{erosion}}\left(t,c\right)+\lambda_{\mathrm{resid}}\left(t,c\right),\\ \lambda_{\mathrm{burst}}\left(t,c\right)\; & =A_{b}\left(c\right)\exp\left(-\frac{t}{\tau_{b}\left(c\right)}\right),\\ \lambda_{\mathrm{diff}}\left(t,c\right)\; & =\frac{A_{d}\left(c\right)}{\sqrt{t+\epsilon }},\\ \lambda_{\mathrm{erosion}}\left(t,c\right)\; & =A_{e}\left(c\right)\sigma \left(k_{e}\left(c\right)\left[t-\tau_{e}\left(c\right)\right]\right),\\ \lambda_{\mathrm{resid}}\left(t,c\right)\; & =\mathrm{softplus}\left(r_{\theta }\left(t,c\right)\right). \end{array}$$

Here, $A_{b}\left(c\right)$, $A_{d}\left(c\right)$, and $A_{e}\left(c\right)$ are positive formulation-dependent amplitudes. The parameter $\tau_{b}\left(c\right)$ controls the burst decay scale, $\tau_{e}\left(c\right)$ controls the onset of the degradation-associated late-stage response, $k_{e}\left(c\right)$ controls its transition rate, and ϵ>0 regularizes the diffusion component at t=0. The subscript “erosion” is retained as the compact mathematical identifier for this late-stage branch. Parameters that must remain positive are generated through Softplus transformations with a small positive offset.

The burst component represents rapid early release associated with drug located at or near the microsphere surface. The diffusion component represents sustained transport through the polymer matrix, interconnected pores, and water-filled channels. The degradation-associated late-stage component represents release acceleration linked to PLGA hydrolysis, pore evolution, matrix relaxation, mass loss, and eventual erosion during the middle and later stages. These coupled effects are represented jointly in the current branch. Paired degradation, mass-loss, and morphology measurements provide the basis for finer process-specific branches. The neural-residual component accounts for systematic dynamics not captured by the three structured functions, including unobserved morphology, nonlinear drug–polymer interactions, or unrecorded formulation differences.

The cumulative release state is defined as

(4) $$\begin{array}{cc} \frac{dR(t)}{dt}\; & =\lambda \left(t,c\right)\left[F_{\infty }\left(c\right)-R\left(t\right)\right],\;R\left(0\right)=0,\\ H(t,c)\; & =\int_{0}^{t}\lambda \left(s,c\right)\;ds,\\ \hat{R}\left(t\right)\; & =F_{\infty }\left(c\right)\left[1-\exp\left(-H(t,c)\right)\right]. \end{array}$$

The quantity H(t,c) is the cumulative release intensity. The three structured components admit closed-form cumulative expressions under Equation (3), while the neural-residual component is accumulated using fixed differentiable quadrature. The model is therefore semi-analytical: it avoids repeated calls to an adaptive ODE solver while retaining numerical integration for the residual component.

**Proposition** **1.** *Assume that $F_{\infty }\left(c\right)\in \left(0,1\right]$ and that $\lambda \left(\cdot ,c\right)\in L_{\mathrm{loc}}^{1}\left(\left[0,\infty \right)\right)$ satisfies λ(t,c)≥0. Then the state equation in Equation* (4) *admits a unique solution. Moreover,*

$$0\leq \hat{R}\left(t\right)\leq F_{\infty }\left(c\right)\leq 1,\;\frac{d\hat{R}\left(t\right)}{dt}\geq 0.$$

*Furthermore, if H(t,c)→∞ as t→∞, then $\hat{R}\left(t\right)\to F_{\infty }\left(c\right)$.*

**Proof.**  The state equation is linear in R(t), and the integrating-factor method gives the analytical solution in Equation (4). Since λ(t,c) is non-negative, H(t,c) is non-decreasing and satisfies H(t,c)≥0. The exponential term therefore lies between zero and one, which establishes non-negativity and boundedness. Differentiating the analytical solution recovers the state equation, whose right-hand side is non-negative. The limiting result follows when the cumulative release intensity diverges. □

The proposition establishes non-negative, monotonic trajectories bounded by the formulation-specific plateau. The forward state map enforces these properties directly.

Training followed a three-stage mechanism-first strategy. During the first stage, the residual rate was fixed to zero, and the formulation encoder, plateau head, burst component, diffusion component, and degradation-associated late-stage component were trained jointly. During the second stage, the residual branch was activated while the learning rates of the structured components were reduced. During the final stage, all components were jointly fine-tuned using a smaller learning rate.

The training objective combines curve-balanced reconstruction, residual control, and parameter regularization:

(5) $$\begin{array}{cc} L_{\mathrm{fit}}\; & =\frac{1}{N}\sum_{i=1}^{N}\frac{1}{n_{i}}\sum_{j=1}^{n_{i}}{\left[R_{ij}-{\hat{R}}_{i}\left(t_{ij}\right)\right]}^{2},\\ q_{\mathrm{resid}}\left(t,c\right)\; & =\lambda_{\mathrm{resid}}\left(t,c\right)\left[F_{\infty }\left(c\right)-\hat{R}\left(t\right)\right],\\ L_{\mathrm{resid}}\; & =\frac{1}{N}\sum_{i=1}^{N}\frac{1}{T_{i}}\int_{0}^{T_{i}}q_{\mathrm{resid}}{\left(t,c_{i}\right)}^{2}\;dt,\\ L\; & =L_{\mathrm{fit}}+\alpha L_{\mathrm{resid}}+\beta L_{\mathrm{reg}}. \end{array}$$

The reconstruction error is first averaged within each formulation and then across formulations, preventing densely sampled profiles from dominating optimization. Residual control acts directly on the residual release flux, so the penalty reflects the neural residual’s contribution to state evolution.

Mechanism attribution is calculated from release fluxes:

(6) $$\begin{array}{cc} q_{m}\left(t,c\right) & =\lambda_{m}\left(t,c\right)\left[F_{\infty }\left(c\right)-\hat{R}\left(t\right)\right],\\ C_{m}\left(T\right) & =\frac{\int_{0}^{T}q_{m}\left(t,c\right)\;dt}{\hat{R}\left(T\right)-\hat{R}\left(0\right)},\;m\in \left\{\mathrm{burst},\mathrm{diff},\mathrm{erosion},\mathrm{resid}\right\}. \end{array}$$

The instantaneous flux $q_{m}\left(t,c\right)$ measures the contribution of component *m* to the current increase in cumulative release, while $C_{m}\left(T\right)$ measures its integrated contribution over [0,T].

**Proposition** **2.** *For any T>0 satisfying $\hat{R}\left(T\right)>\hat{R}\left(0\right)$, the flux-based contributions satisfy*

$$C_{m}\left(T\right)\geq 0,\;\sum_{m}C_{m}\left(T\right)=1.$$

**Proof.**  The total release rate is the sum of the mechanism-specific rates. Consequently, the sum of the mechanism fluxes equals the derivative of the cumulative release state. Integrating over [0,T] gives the total release increment. Dividing each integrated flux by this increment proves non-negativity and conservation of the contributions. □

The four contributions are reported as model-attributed release-component summaries. Proposition 2 establishes non-negative, conservative accounting of the predicted release increment. Orthogonal mechanism measurements assess physical process alignment, while the real-data attribution supports comparative and hypothesis-generating analysis. During inference, the formulation vector is encoded once, the mechanism-specific parameters are generated from the latent context, cumulative intensities are evaluated at the requested query times, and the state map produces the release profile. The same forward computation yields mechanism fluxes and the attribution vector.

### 4.3. Synthetic Mechanism-Recovery Benchmark

Component-level ground-truth labels were available in the controlled synthetic setting. The compact label “erosion” is retained in the synthetic class names and plotted figure labels. A synthetic benchmark was therefore constructed to evaluate whether MC-NODE can recover known mechanism contributions when the generating components are controlled. The benchmark was designed to test empirical recoverability under specified generators.

Synthetic curves were generated using formulation feature vectors sampled from the real dataset and time grids matched to observed release schedules. Each synthetic curve was assigned to one of four scenarios: burst-dominant, diffusion-dominant, erosion-dominant, or mixed. In the three dominant scenarios, the corresponding mechanism was required to contribute at least 55% of the integrated release. In the mixed scenario, no structured mechanism was allowed to exceed 45%. The residual contribution was constrained to remain within a moderate range so that unresolved dynamics were present but did not dominate the profile.

Three conditions were considered. Condition A used a matched generator without observation noise. Condition B used the same generator with 50% temporal observation retention and Gaussian observation noise with a standard deviation of 0.03 on the release fraction scale. Condition C used a mildly mismatched generator with the same sparse and noisy observation setting. The mismatched generator preserved the broad roles of burst, diffusion, and erosion but used temporal forms that were not identical to the MC-NODE branches. This condition tested whether the model could recover approximate mechanism contributions when the assumed structure was imperfect.

Each condition contained balanced burst-dominant, diffusion-dominant, erosion-dominant, and mixed scenarios. The generated curves were split into training, validation, and test subsets using stratified sampling. The test subset contained 180 curves for each condition. The models were trained using the same architecture and optimization settings as in the real-data experiments. The model observed the sparse and noisy values during training when applicable, while evaluation was performed against the clean generating curve and the known integrated mechanism contributions.

Synthetic recovery was evaluated using four metrics. Curve RMSE measured prediction accuracy on a dense time grid. Contribution MAE measured the mean absolute difference between predicted and true mechanism contributions in percentage points. Dominant-class accuracy measured whether the predicted dominant release scenario matched the true scenario. Mean Spearman correlation measured the rank association between predicted and true contributions across mechanisms and test curves. These metrics jointly assessed whether a model could both fit the total release profile and recover the underlying contribution structure.

### 4.4. Comparison Methods and Ablation Design

The comparison set was organized to separate descriptive curve fitting from formulation-level prediction. Descriptive kinetic references were first included to show how well classical equations could fit individual release curves when the target trajectory was available. Formulation-level predictors were then grouped according to their inductive bias, including kinetic-parameter predictors, pointwise predictors, continuous-time predictors, and a structure-matched constrained baseline. This design follows the common-task principle of evaluating representative model families under a shared protocol [36] and allows the effects of kinetic parameterization, nonlinear formulation modelling, continuous-time dynamics, structural constraints, and mechanism decomposition to be assessed separately.

The descriptive kinetic references comprised the first-order, Higuchi, Korsmeyer–Peppas, and Weibull equations. Their parameters were fitted independently to each observed release curve using nonlinear least squares. These models served as retrospective fitting references because their parameters came directly from the target release profile. Predictor ranking and paired statistical comparison were restricted to formulation-level methods.

To provide a same-task comparison with classical kinetic modelling, two formulation-to-kinetic-parameter predictors were additionally constructed. For each outer training split, the Korsmeyer–Peppas and Weibull equations were first fitted to the training curves only. The fitted kinetic parameters were then used as supervised targets for LightGBM regressors trained from the ten formulation descriptors. At test time, the trained regressors predicted the kinetic parameters from formulation descriptors, and the corresponding kinetic equation was used to generate the release curve at the test observation times. These baselines were denoted KP-Param-LightGBM and Weibull-Param-LightGBM. These predictors generated test trajectories exclusively from formulation descriptors and were evaluated as formulation-level predictors. This distinction separates direct retrospective kinetic fitting from predictive use of kinetic equations on unseen formulations.

The pointwise formulation predictors comprised LightGBM, a time-conditioned MLP, and FT-Transformer. LightGBM was selected as a strong tree-based baseline because gradient-boosted trees have demonstrated competitive performance in polymeric long-acting injectable modelling [10]. The MLP and FT-Transformer represented feed-forward and attention-based models for heterogeneous tabular data [37]. Each model received the ten formulation predictors together with *t* and log(1+t) and directly estimated the cumulative release fraction at the query time. The pointwise neural predictors used linear output layers, and their raw outputs were evaluated directly for range and monotonicity violations.

The continuous-time predictor category included an unconstrained Neural ODE and a Penalty-Constrained MLP. The Neural ODE generated the release trajectory from a formulation-conditioned neural vector field through numerical integration [23]. The Penalty-Constrained MLP used the same architecture as the time-conditioned MLP but added monotonicity and range penalties to the training objective. The monotonicity penalty weight was set to $\gamma_{\mathrm{mono}}=1.0$, and the range penalty weight was set to $\gamma_{\mathrm{range}}=1.0$ after validation tuning. This baseline encoded physical consistency through the loss function, complementing the state-structured comparison.

SingleRate-NODE was constructed as the closest architectural baseline. It retained the formulation encoder, plateau head, positive-rate transformation, and semi-analytical state map of MC-NODE, while replacing the burst, diffusion, degradation-associated late-stage, and residual branches with one time-conditioned neural release-rate function. Its parameter count was controlled within 10% of the parameter count of MC-NODE. The comparison between SingleRate-NODE and MC-NODE isolates the contribution of mechanism decomposition while preserving the continuous-time formulation, positive-rate constraint, formulation-dependent plateau, and bounded state representation. In the ablation table, this model is also reported as MC-NODE without mechanism decomposition.

Five ablation variants were derived from the complete MC-NODE model. The first variant removed mechanism decomposition by replacing the four release branches with the single positive-rate function used in SingleRate-NODE. The second removed the neural-residual branch and retained only the structured burst, diffusion, and degradation-associated late-stage components. The third activated all release branches from the beginning of training and removed the mechanism-first schedule. The fourth retained the residual branch but removed residual-flux regularization. The fifth fixed the final release plateau to one and removed formulation-dependent plateau prediction. These ablations were designed to test whether prediction performance and mechanism recovery were attributable to the proposed release decomposition, residual pathway, optimization schedule, residual control, and plateau head.

All formulation-level predictors used the same ten formulation descriptors, the same temporal query variables, the same experimental partitions, and the same target release values. Neural methods were evaluated using the same five random seeds. Hyperparameters were selected exclusively from validation data under an equal search budget, followed by one-time evaluation on each held-out test partition. The kinetic-parameter predictors, pointwise predictors, continuous-time predictors, SingleRate-NODE, and MC-NODE were included in the formulation-level statistical comparison. The descriptive kinetic references were reported separately because they used the observed target curve during parameter fitting.

### 4.5. Implementation Details

MC-NODE was implemented in PyTorch with a 64-dimensional formulation context. The formulation encoder contained three fully connected layers with widths of 128, 128, and 64. Each structured mechanism used a separate parameter head containing one 32-unit hidden layer. The plateau head also used one 32-unit hidden layer followed by a sigmoid output. GELU activation, layer normalization, and a dropout rate of 0.10 were applied to the neural modules.

The neural-residual branch received the formulation context, *t*, and log(1+t). It contained two hidden layers with 64 units and used a Softplus output to ensure a non-negative residual rate. Positive amplitudes, time scales, and transition parameters in the structured branches were also produced through Softplus transformations. The burst, diffusion, and degradation-associated late-stage contributions were accumulated using their analytical integrals, while the residual contribution was calculated using 16-point Gauss–Legendre quadrature. The diffusion offset was set to $\epsilon =10^{-6}$.

Training followed the three-stage mechanism-first schedule. During the first stage, the residual branch was disabled and the structured mechanism components were trained for up to 300 epochs with a learning rate of $10^{-3}$. During the second stage, the residual branch was activated for up to 150 epochs with a learning rate of $5\times 10^{-4}$, while the learning rate of the structured components was reduced to $10^{-4}$. During the final stage, all components were jointly fine-tuned for up to 150 epochs using a learning rate of $10^{-4}$.

AdamW was used with a batch size of 16 complete release curves, gradient clipping at a norm of 5, and early stopping after 40 epochs without improvement in the curve-balanced validation RMSE. The residual regularization coefficient was set to $\alpha =10^{-2}$, and the weight regularization coefficient was set to $\beta =10^{-5}$ based on validation performance. Each learning-based model received 30 hyperparameter trials, and the final configuration was selected using validation data only. All neural models were trained using five random seeds. SingleRate-NODE and the neural baselines used comparable representation dimensions and training budgets to support controlled comparison.

### 4.6. Evaluation Protocol

The primary real-data experiment used DOI-grouped five-fold cross-validation. All formulations originating from the same publication were assigned to the same outer fold. Within each outer training split, the validation subset was formed using the same publication-level grouping and accounted for approximately 10% of the training publications. Feature transformations, hyperparameter selection, and early stopping used only the outer training and validation partitions. Predictions from the five held-out folds were concatenated to obtain one out-of-fold prediction for every retained formulation. Each random seed therefore produced one complete set of out-of-fold predictions across the 321 release curves.

Drug-level extrapolation was evaluated using drug-grouped five-fold cross-validation. All formulations associated with the same drug identity were assigned to the same fold, ensuring that the tested drug identity was absent from training. This protocol evaluates identity-level drug extrapolation; fingerprint-cluster-grouped validation separately evaluates chemical dissimilarity.

Sparse-time robustness was evaluated by retaining 25%, 50%, 75%, or 100% of the observations in each training curve while keeping every test curve complete. The first and final observations of each training curve were always retained. The remaining observations were sampled from early, middle, and late portions of the release interval to avoid retaining only one release stage. The same outer folds, validation folds, and random seeds were used across compared methods.

### 4.7. Additional Robustness, Extrapolation, and Diagnostic Protocols

Dependence on the structured temporal bases was evaluated by replacing the default burst, diffusion, and degradation-associated late-stage functions individually and jointly. The alternative rates were defined as follows:

$$\lambda_{\mathrm{burst}}^{\mathrm{alt}}\left(t,c\right)=A_{b}\left(c\right)\exp\;\left[-{\left(\frac{t}{\tau_{b}\left(c\right)}\right)}^{p_{b}\left(c\right)}\right]$$

$$\lambda_{\mathrm{diff}}^{\mathrm{alt}}\left(t,c\right)=\frac{A_{d}\left(c\right)}{{\left[t+\epsilon \right]}^{p_{d}\left(c\right)}}$$

$$\lambda_{\mathrm{late}}^{\mathrm{alt}}\left(t,c\right)=A_{e}\left(c\right)\exp\;\left\{-\exp[-k_{e}\left(c\right)\left(t-\tau_{e}\left(c\right)\right)]\right\},$$

where, $p_{b}\left(c\right)>0$ and $0<p_{d}\left(c\right)<1$ were formulation-dependent, and all amplitudes, time scales, and transition rates retained the positive parameterization used by the default model. Every variant retained the same plateau and state map, DOI-grouped folds, five random seeds, and training budget. Prediction was evaluated on the real dataset, while contribution recovery was evaluated under synthetic Condition C. Dominant-component agreement was the percentage of real test curves for which the largest integrated component matched that of the default MC-NODE under the same fold and seed.

Optimization sensitivity was assessed using early residual activation (200/200/200 epochs), the default schedule (300/150/150), late residual activation (400/100/100), one-half and two-times learning rates, and a validation-plateau transition rule capped at 600 epochs. Residual regularization was varied over $\alpha \in \{0,10^{-3},10^{-2},10^{-1}\}$ with $\beta =10^{-5}$, and weight regularization was varied over $\beta \in \{0,10^{-6},10^{-5},10^{-4}\}$ with $\alpha =10^{-2}$. One coefficient was varied at a time.

Chemical extrapolation was evaluated from the reported SMILES representations using 2048-bit Morgan fingerprints with radius 2 and Tanimoto similarity. Drugs were grouped by Butina clustering on Tanimoto distance, 1−similarity, using a cutoff of 0.55. This produced 31 clusters, including 12 singletons; the largest cluster contained eight drugs. Complete clusters were assigned as indivisible units to five folds by greedy balancing of curve count, followed by drug and cluster counts. The test folds contained 6–7 clusters, 17–18 drugs, and 61–68 curves. For every test drug, the maximum Tanimoto similarity to a training drug was calculated and grouped into low [0.18,0.41), medium [0.41,0.57), and high [0.57,0.86] similarity strata. A standardized distance based on molecular weight, topological polar surface area, and LogP was also grouped into inside-distribution [0.40,1.35), moderate-distance [1.35,2.25), and high-distance [2.25,5.10] strata.

Endpoint dependence in sparse-time training was tested at 25% and 50% retention using five sampling rules: Anchored retained both endpoints, Random imposed no endpoint constraint, No-first retained only the final endpoint, No-final retained only the first endpoint, and Interior-only sampled exclusively from internal observations. Sampling masks were shared by the compared models within each fold and seed, and all test trajectories remained complete.

Individual uncertainty was quantified with the five-seed ensemble and fold-internal conformal calibration. For S=5 seed models, the ensemble mean and dispersion were

$$\mu_{i}\left(t\right)=\frac{1}{S}\sum_{s=1}^{S}{\hat{R}}_{i}^{\left(s\right)}\left(t\right),\;\sigma_{i}\left(t\right)=\sqrt{\frac{1}{S-1}\sum_{s=1}^{S}{\left[{\hat{R}}_{i}^{\left(s\right)}\left(t\right)-\mu_{i}\left(t\right)\right]}^{2}}$$

For each validation trajectory, the nonconformity score was

$$s_{i}=\max_{j}\frac{|R_{ij}-\mu_{i}\left(t_{ij}\right)|}{\sigma_{i}\left(t_{ij}\right)+\epsilon_{u}}$$

where $\epsilon_{u}=10^{-3}$ prevents division by zero. The finite-sample-corrected 90% quantile $q_{0.90}$ of the calibration scores defined the simultaneous test band

$$I_{i}\left(t\right)=\left[\mu_{i}\left(t\right)-q_{0.90}\left(\sigma_{i}\left(t\right)+\epsilon_{u}\right),\;\mu_{i}\left(t\right)+q_{0.90}\left(\sigma_{i}\left(t\right)+\epsilon_{u}\right)\right]\cap \left[0,1\right].$$

After checkpoint selection, calibration scores were computed from group-disjoint validation trajectories within each outer training split, and the corresponding outer test fold remained isolated. The five chemical-cluster folds used 24, 23, 26, 25, and 24 calibration trajectories, respectively. Evaluation reported pointwise coverage, whole-trajectory coverage, mean and median band width, and the Spearman association between band width and curve RMSE.

Descriptor diagnostics used formulation-level Spearman correlations, variance inflation factors (VIFs), and the condition number of the standardized design matrix. Permutation importance was calculated at the formulation level so that all time points of one curve retained a consistent shuffled descriptor. Importance was the increase in out-of-fold RMSE for the complete trajectory and for the early, middle, and late regions. Correlated descriptor groups were additionally permuted together. Two reduced variants removed the lowest-ranked descriptor and then the two lowest-ranked descriptors.

MC-NODE and SingleRate-NODE were benchmarked on a single NVIDIA GeForce RTX 4080 SUPER with 16 GB memory using Python 3.11.9, PyTorch 2.3.1, and CUDA 12.1. Batch size was 1 with a fixed grid of 100 query times. Latency was averaged over 1000 CUDA-synchronized runs after 100 warm-up runs. Parameter count, training time per fold, latency per formulation, and peak GPU memory were recorded.

A retrospective formulation-matched orthogonal consistency analysis was conducted using 18 formulations for which a release trajectory and at least one independent experimental mechanism indicator were available. The matched formulations were drawn from four source studies [38,39,40,41], contributing 5, 5, 4, and 4 formulations, respectively. Fourteen formulations were evaluated using DOI-grouped out-of-fold predictions, and four were evaluated using locked external inference; only the 37 °C real-time condition was used from Zolnik et al. The indicators comprised degradation rate, polymer molecular-weight loss, pore evolution, polymer mass loss, or a diffusion-related measurement. Release trajectories alone determined training and model selection, and the orthogonal measurements served exclusively as independent evaluation targets. Spearman correlation quantified associations between the degradation-associated late-stage contribution and degradation-related indicators and between the diffusion contribution and the diffusion indicator. Late-stage onset MAE was calculated over the 15 formulations with paired predicted and experimentally derived onset times. Dominant-process agreement was the proportion of formulations for which the largest structured contribution matched the experimental process assignment. Curve-balanced RMSE was also reported for the 18 matched release trajectories.

### 4.8. Evaluation Metrics and Statistical Analysis

Prediction accuracy was evaluated at the level of complete release curves. Let $R_{ij}$ and ${\hat{R}}_{ij}$ denote the observed and predicted release fractions for formulation *i* at its *j*th observation time. Curve-balanced MAE and RMSE were calculated as

(7) $$\mathrm{MAE}=\frac{1}{N}\sum_{i=1}^{N}\frac{1}{n_{i}}\sum_{j=1}^{n_{i}}\left|R_{ij}-{\hat{R}}_{ij}\right|,\;\mathrm{RMSE}=\frac{1}{N}\sum_{i=1}^{N}\sqrt{\frac{1}{n_{i}}\sum_{j=1}^{n_{i}}{\left(R_{ij}-{\hat{R}}_{ij}\right)}^{2}},$$

where *N* is the number of test curves and $n_{i}$ is the number of observations in curve *i*. Averaging within each curve before averaging across formulations prevents densely sampled profiles from dominating the evaluation. The coefficient of determination was computed using all out-of-fold test observations pooled within each random seed.

Differences in overall curve shape were measured using the normalized integrated absolute error:

(8) $$\mathrm{nIAE}=\frac{1}{N}\sum_{i=1}^{N}\frac{\int_{t_{i1}}^{T_{i}}\left|R_{i}\left(t\right)-{\hat{R}}_{i}\left(t\right)\right|\;dt}{T_{i}-t_{i1}},$$

where $T_{i}$ denotes the final observation time. The integral was approximated using the trapezoidal rule over the original observation times. The absolute error in the estimated time to 50% release was calculated only for curves that reached a release fraction of 0.50 during the observation period. The observed $t_{50}$ and predicted $t_{50}$ values were obtained by linear interpolation between adjacent time points.

Stage-wise prediction errors were additionally calculated to examine whether model performance differed across release phases. For each test curve, the observation time was normalized as

$$\tau =\frac{t-t_{i1}}{T_{i}-t_{i1}},$$

where $t_{i1}$ and $T_{i}$ denote the first and final observation times of curve *i*. Observations were divided into early release (0≤τ<0.2), middle release (0.2≤τ<0.7), and late release (0.7≤τ≤1.0) regions. RMSE was calculated within each region using the same out-of-fold predictions as the main evaluation. Curves without observations in a given region were omitted from that region-specific calculation.

Physical consistency was assessed at the curve level. A monotonicity violation was recorded when a predicted release value decreased between two consecutive query times by more than a numerical tolerance $\varepsilon =10^{-6}$. A range violation was recorded when any predicted value was below zero or above one. The corresponding violation rates were defined as the proportions of test curves containing at least one such event.

Terminal-release-stratified analysis was performed to evaluate whether the formulation-dependent plateau improved prediction across different final-release levels. Test curves were grouped according to the observed final cumulative release fraction into low terminal release (0.60≤R(T)<0.75), medium terminal release (0.75≤R(T)<0.90), and high terminal release (R(T)≥0.90). Within each group, full-profile RMSE, nIAE, late-stage RMSE, final-release MAE, and final-release bias were calculated using the same out-of-fold predictions as the primary evaluation. Late-stage RMSE was calculated over the normalized time interval 0.70≤τ≤1.00. Final-release MAE and bias were defined as

$$\mathrm{Final-release}\;\mathrm{MAE}=\frac{1}{N_{g}}\sum_{i\in g}\left|{\hat{R}}_{i}\left(T_{i}\right)-R_{i}\left(T_{i}\right)\right|,\;\mathrm{Bias}=\frac{1}{N_{g}}\sum_{i\in g}\left[{\hat{R}}_{i}\left(T_{i}\right)-R_{i}\left(T_{i}\right)\right],$$

where *g* denotes a terminal-release group and $N_{g}$ is the number of curves in that group. A positive bias indicates overprediction of the final cumulative release fraction.

For chemical applicability-domain analysis, curve RMSE was related to maximum test-to-training Tanimoto similarity and to standardized physicochemical distance using Spearman correlation. VIF was calculated as $1/(1-R_{j}^{2})$ for each descriptor regressed on the remaining descriptors. Permutation importance was expressed as ΔRMSE relative to the unpermuted out-of-fold predictions. For the 90% prediction bands, whole-trajectory coverage required every observed value of a curve to lie inside the simultaneous band.

For the main prediction experiments, each random seed produced one complete out-of-fold prediction after concatenating the five test folds. Results were reported as the mean and standard deviation across five random seeds. For statistical comparison, the error of each test curve was first averaged across seeds. MC-NODE and each predictive baseline were then compared using the paired Wilcoxon signed-rank test over matched release curves. The Holm procedure was applied to correct for multiple comparisons, and an adjusted *p*-value below 0.05 was considered statistically significant. Complete release curves were treated as the statistical units throughout the analysis [36].

## 5. Conclusions

This study proposed MC-NODE, a mechanism-decomposed neural differential model for PLGA microsphere drug release prediction and model-attributed release-component analysis. The model combines formulation-context encoding, non-negative release-rate decomposition, a formulation-dependent plateau, and a semi-analytical state map to generate continuous, monotonic, and bounded cumulative release trajectories. The release process is represented through four components for burst release, diffusion, degradation-associated late-stage release, and neural residuals, and the predicted release increment is further decomposed through flux-based attribution.

The main prediction experiments demonstrated that MC-NODE achieved the strongest formulation-level performance on the literature-curated PLGA dataset. Under DOI-grouped five-fold cross-validation, MC-NODE obtained an RMSE of 0.094±0.005, an MAE of 0.069±0.004, an $R^{2}$ of 0.854±0.018, and an nIAE of 0.061±0.004. All 321 out-of-fold test curves satisfied the monotonicity and range criteria, confirming that the structure-preserving state representation generated physically admissible cumulative release profiles directly.

The additional analyses clarified where the main advantages of MC-NODE arose. The ablation study showed that mechanism decomposition, the neural-residual branch, mechanism-first optimization, residual control, and the formulation-dependent plateau played complementary roles. The stage-wise analysis indicated that MC-NODE improved prediction most clearly in the early and late release regions, where burst behavior, terminal release levels, and degradation-related dynamics are more influential. The terminal-release-stratified analysis further showed that the learned plateau reduced final-release overprediction in low and medium terminal-release profiles, whereas the fixed-plateau assumption became less harmful when curves approached complete release.

The synthetic mechanism-recovery benchmark provided controlled evidence that MC-NODE could recover known contribution labels more accurately than ablated variants under clean, sparse, noisy, and mildly mismatched settings. The retrospective formulation-matched analysis provided direct experimental consistency across 18 formulations: component–indicator correlations ranged from ρ=0.476 to 0.738, dominant-process agreement was 83.3%, late-stage onset MAE was 4.95 days (median absolute error, 4.20 days), and matched release-trajectory RMSE was 0.108±0.020. The release-component outputs were therefore reported as experimentally supported model-attributed summaries for comparative and hypothesis-generating analysis. The attribution analysis indicated that diffusion was the dominant model-attributed component in the largest proportion of release curves, followed by burst release and the degradation-associated late-stage component, while the residual component remained comparatively limited.

Drug-grouped and sparse-time evaluations further showed that MC-NODE retained the lowest absolute trajectory-level errors under unseen drug identities and limited temporal observations. The additional robustness analyses showed stable prediction under single changes to the structured temporal forms and under moderate changes to the training schedule and regularization. Fingerprint-cluster validation yielded an RMSE of 0.130±0.011, with error increasing as chemical similarity decreased. Simultaneous 90% trajectory bands achieved 90.7±1.8% coverage under DOI-grouped validation and widened under chemical OOD. Descriptor diagnostics identified PLGA molecular weight as most influential overall and during late release, while particle size and drug loading were most influential during early release. These results suggest that mechanism-decomposed neural differential modelling can support retrospective formulation comparison, candidate prioritization, and hypothesis generation for PLGA microsphere release studies. Broader synchronized experimental datasets, standardized protocol metadata, controlled prospective studies, and paired in vitro–in vivo measurements can extend the framework to external datasets and other polymeric delivery systems.

## Figures and Tables

**Table 1:** DOI-grouped prediction performance (mean ± SD).

Model	MAE ↓	RMSE ↓	$R^{2}$↑	nIAE ↓	$t_{50}$ Error (Day) ↓
Descriptive kinetic references					
First-order	0.082±0.010	0.108±0.012	0.794±0.041	0.076±0.010	4.70±0.62
Higuchi	0.074±0.009	0.099±0.011	0.821±0.037	0.068±0.008	4.15±0.58
Korsmeyer–Peppas	0.061±0.007	0.083±0.009	0.881±0.029	0.056±0.007	3.21±0.47
Weibull	0.051±0.006	0.070±0.008	0.916±0.023	0.046±0.006	2.68±0.41
Kinetic-parameter predictors					
KP-Param-LightGBM	0.087±0.006	0.117±0.008 ***	0.789±0.031	0.082±0.006	5.28±0.61
Weibull-Param-LightGBM	0.080±0.005	0.108±0.007 **	0.817±0.027	0.074±0.005	$\underline{3.92\pm 0.48}$
Pointwise predictors					
LightGBM	0.094±0.005	0.127±0.007 ***	0.762±0.028	0.089±0.006	6.32±0.71
Time-conditioned MLP	0.088±0.006	0.118±0.008 ***	0.785±0.031	0.083±0.006	5.53±0.64
FT-Transformer	0.084±0.005	0.113±0.006 **	0.801±0.025	0.078±0.005	5.01±0.55
Continuous-time predictors					
Neural ODE	0.081±0.006	0.109±0.007 **	0.811±0.026	0.075±0.006	4.78±0.57
Penalty-Constrained MLP	0.078±0.005	0.105±0.006 *	0.823±0.023	0.072±0.005	4.43±0.49
Structure-matched baseline					
SingleRate-NODE	$\underline{0.074\pm 0.004}$	$\underline{0.100\pm 0.005}$ *	$\underline{0.837\pm 0.020}$	$\underline{0.067\pm 0.004}$	3.88±0.43
Proposed method					
MC-NODE	0.069±0.004	0.094±0.005	0.854±0.018	0.061±0.004	3.97±0.40

↓ and ↑ indicate that lower and higher values, respectively, represent better performance. Bold and underlined values indicate the best and second-best formulation-level results, respectively. * *p* < 0.05, ** *p* < 0.01, and *** *p* < 0.001; Holm-adjusted Wilcoxon tests versus MC-NODE.

**Table 2:** Temporal-form sensitivity.

Variant	RMSE	nIAE	Residual (%)	Agreement (%)	Contribution MAE	Accuracy (%)
Default	0.0940±0.0050	0.0610±0.0040	12.60±2.50	100.0±0.0	8.90±1.20	82.4±3.2
Alternative burst	0.0955±0.0056	0.0622±0.0046	13.46±2.38	92.8±2.3	9.80±1.35	79.6±3.6
Alternative diffusion	0.0937±0.0054	0.0612±0.0044	14.92±0.87	90.9±2.8	10.20±1.45	78.8±3.9
Alternative late-stage	0.0959±0.0059	0.0625±0.0048	13.94±2.36	91.6±2.7	10.50±1.50	78.1±4.0
All alternatives	0.0981±0.0065	0.0648±0.0053	17.36±4.03	84.0±3.5	12.60±1.75	72.5±4.8

**Table 3:** Training-schedule sensitivity.

Configuration	Stage Epochs	RMSE	nIAE	Residual (%)	Epochs Used
Early activation	200/200/200	0.0968±0.0060	0.0635±0.0048	18.68±1.13	512±28
Default	300/150/150	0.0940±0.0050	0.0610±0.0040	12.60±2.50	548±22
Late activation	400/100/100	0.0955±0.0057	0.0622±0.0045	10.18±4.10	565±18
Half learning rate	300/150/150	0.0951±0.0054	0.0618±0.0044	11.80±2.74	590±12
Double learning rate	300/150/150	0.0992±0.0080	0.0650±0.0060	15.50±4.56	487±45
Adaptive transition	validation plateau	0.0938±0.0053	0.0608±0.0042	13.12±1.27	504±30

**Table 4:** Regularization sensitivity.

Configuration	MAE	RMSE	nIAE	Residual (%)	Contribution MAE	Accuracy (%)
$\alpha =0,\;\beta =10^{-5}$	0.0680±0.0050	0.0970±0.0070	0.0640±0.0050	34.10±5.80	13.20±1.90	70.8±5.0
$\alpha =10^{-3},\;\beta =10^{-5}$	0.0690±0.0045	0.0948±0.0055	0.0618±0.0045	20.50±3.60	10.40±1.50	78.4±4.0
$\alpha =10^{-2},\;\beta =10^{-5}$	0.0690±0.0040	0.0940±0.0050	0.0610±0.0040	12.60±2.50	8.90±1.20	82.4±3.2
$\alpha =10^{-1},\;\beta =10^{-5}$	0.0720±0.0051	0.0987±0.0064	0.0648±0.0051	5.76±4.01	10.80±1.60	76.9±4.2
$\alpha =10^{-2},\;\beta =0$	0.0698±0.0049	0.0954±0.0061	0.0623±0.0049	13.72±3.71	9.40±1.40	80.6±3.7
$\alpha =10^{-2},\;\beta =10^{-6}$	0.0693±0.0044	0.0946±0.0055	0.0615±0.0044	13.00±3.60	9.10±1.30	81.5±3.5
$\alpha =10^{-2},\;\beta =10^{-4}$	0.0705±0.0047	0.0963±0.0059	0.0629±0.0047	11.86±4.01	9.80±1.40	79.4±3.8

**Table 5:** Chemical-cluster holdout.

Model	MAE	RMSE	$R^{2}$	nIAE	$t_{50}$ Error (Day)
FT-Transformer	0.111±0.009	0.151±0.013	0.690±0.035	0.101±0.009	6.40±0.58
SingleRate-NODE	0.101±0.008	0.137±0.011	0.728±0.031	0.091±0.008	5.87±0.46
MC-NODE	0.096±0.008	0.130±0.011	0.751±0.029	0.085±0.007	5.98±0.48

**Table 6:** Performance by Tanimoto similarity.

Stratum	Tanimoto Interval	Drugs	Curves	RMSE	nIAE
Low	[0.18,0.41)	28	91	0.149±0.012	0.099±0.009
Medium	[0.41,0.57)	30	106	0.128±0.010	0.084±0.007
High	[0.57,0.86]	31	124	0.110±0.009	0.071±0.006

**Table 7:** High-error physicochemical regions.

Region	Definition	Drugs	Curves	RMSE	nIAE
High MW/high TPSA	MW >500, TPSA >100	8	29	0.158	0.104
Highly lipophilic/low TPSA	LogP >4.0, TPSA <45	7	25	0.154	0.101
Very hydrophilic outliers	LogP <0, TPSA >85	6	21	0.162	0.108

**Table 8:** Sparse-sampling sensitivity.

Model	Retention	Scenario	RMSE	nIAE	ΔRMSE vs. Anchored	ΔnIAE vs. Anchored
MC-NODE	25%	Anchored	0.1160±0.0123	0.0770±0.0076	0.0%	0.0%
MC-NODE	25%	Random	0.1285±0.0135	0.0860±0.0084	10.8%	11.7%
MC-NODE	25%	No-first	0.1238±0.0130	0.0823±0.0081	6.7%	6.9%
MC-NODE	25%	No-final	0.1326±0.0142	0.0894±0.0090	14.3%	16.1%
MC-NODE	25%	Interior-only	0.1398±0.0150	0.0948±0.0096	20.5%	23.1%
MC-NODE	50%	Anchored	0.1046±0.0087	0.0693±0.0055	0.0%	0.0%
MC-NODE	50%	Random	0.1118±0.0095	0.0747±0.0061	6.9%	7.8%
MC-NODE	50%	No-first	0.1115±0.0089	0.0721±0.0059	6.6%	4.0%
MC-NODE	50%	No-final	0.1150±0.0100	0.0770±0.0064	9.9%	11.1%
MC-NODE	50%	Interior-only	0.1204±0.0108	0.0808±0.0069	15.1%	16.6%
SingleRate-NODE	25%	Anchored	0.1280±0.0129	0.0870±0.0083	0.0%	0.0%
SingleRate-NODE	25%	Random	0.1380±0.0140	0.0950±0.0091	7.8%	9.2%
SingleRate-NODE	25%	No-first	0.1340±0.0136	0.0920±0.0088	4.7%	5.7%
SingleRate-NODE	25%	No-final	0.1420±0.0148	0.0990±0.0096	10.9%	13.8%
SingleRate-NODE	25%	Interior-only	0.1480±0.0154	0.1040±0.0100	15.6%	19.5%
SingleRate-NODE	50%	Anchored	0.1134±0.0091	0.0770±0.0060	0.0%	0.0%
SingleRate-NODE	50%	Random	0.1210±0.0100	0.0830±0.0066	6.7%	7.8%
SingleRate-NODE	50%	No-first	0.1180±0.0097	0.0805±0.0064	4.1%	4.5%
SingleRate-NODE	50%	No-final	0.1250±0.0105	0.0865±0.0070	10.2%	12.3%
SingleRate-NODE	50%	Interior-only	0.1310±0.0113	0.0910±0.0075	15.5%	18.2%

**Table 9:** Trajectory-band calibration.

Setting	Pointwise Coverage	Trajectory Coverage	Mean Width	Median Width	Error–Width ρ	High-UQ RMSE
DOI-grouped CV	97.3±0.9%	90.7±1.8%	0.162±0.012	0.148±0.011	0.61±0.05	0.154±0.012
Chemical-cluster OOD	96.1±1.2%	89.6±2.4%	0.218±0.018	0.201±0.016	0.66±0.06	0.196±0.017

**Table 10:** Descriptor diagnostics and permutation importance.

Descriptor	VIF	Overall ΔRMSE	Early ΔRMSE	Middle ΔRMSE	Late ΔRMSE
Drug molecular weight	1.58	0.0044±0.0012	0.0032±0.0011	0.0041±0.0012	0.0055±0.0015
TPSA	1.72	0.0050±0.0013	0.0045±0.0012	0.0037±0.0011	0.0060±0.0016
LogP	1.66	0.0058±0.0015	0.0056±0.0015	−0.0002±0.0005	0.0074±0.0018
PLGA molecular weight	1.29	0.0095±0.0018	0.0038±0.0012	0.0082±0.0017	0.0131±0.0022
LA:GA ratio	1.18	0.0078±0.0016	0.0034±0.0011	0.0067±0.0015	0.0106±0.0020
Initial drug-to-polymer ratio	2.78	0.0067±0.0015	0.0090±0.0018	0.0046±0.0013	0.0049±0.0014
Particle size	1.42	0.0091±0.0018	0.0127±0.0021	0.0062±0.0015	0.0070±0.0017
Drug loading	3.36	0.0087±0.0017	0.0120±0.0020	0.0057±0.0014	0.0050±0.0014
Encapsulation efficiency	2.41	0.0061±0.0014	0.0073±0.0016	0.0049±0.0013	0.0052±0.0014
Solubility-enhancer concentration	1.36	0.0022±0.0009	0.0031±0.0010	0.0013±0.0007	0.0017±0.0008

**Table 11:** Reduced-descriptor models.

Model	MAE	RMSE	$R^{2}$	nIAE
Full MC-NODE (10)	0.0690±0.0040	0.0940±0.0050	0.854±0.018	0.0610±0.0040
w/o enhancer (9)	0.0694±0.0043	0.0946±0.0053	0.852±0.019	0.0615±0.0042
w/o enhancer + drug MW (8)	0.0715±0.0048	0.0970±0.0058	0.845±0.021	0.0637±0.0047

**Table 12:** Model complexity and runtime.

Model	Parameters	Training/Fold (min)	Latency (ms)	Peak GPU (GB)
SingleRate-NODE	42,912	4.80±0.25	0.84±0.05	0.42±0.02
MC-NODE	44,176	6.30±0.32	1.21±0.07	0.48±0.02

**Table 13:** Orthogonal consistency.

Indicator	Component	Metric	*N*
Degradation rate	Late-stage	ρ=0.555	13
MW loss	Late-stage	ρ=0.527	13
Pore evolution	Late-stage	ρ=0.738	8
Mass loss	Late-stage	ρ=0.476	8
Diffusion indicator	Diffusion	ρ=0.599	8
Onset	Late-stage	MAE =4.95 d	15
Dominant process	Structured	83.3% (15/18)	18
Trajectory	–	RMSE =0.108±0.020	18

**Table 14:** MC-NODE ablation results (mean ± SD).

Model Variant	MAE ↓	RMSE ↓	$R^{2}$↑	nIAE ↓	Residual Contribution (%)
w/o Mechanism Decomposition	0.074±0.004	0.100±0.005	0.837±0.020	0.067±0.004	N/A
w/o Neural Residual	0.073±0.005	0.099±0.006	0.840±0.021	0.066±0.005	0.0
w/o Mechanism-First Training	0.072±0.005	0.098±0.006	0.844±0.021	0.065±0.005	24.8±4.3
w/o Residual Control	0.068±0.005	$\underline{0.097\pm 0.007}$	$\underline{0.846\pm 0.023}$	$\underline{0.064\pm 0.005}$	34.1±5.8
Fixed Plateau	0.076±0.006	0.103±0.007	0.829±0.025	0.070±0.006	18.3±3.7
**MC-NODE**	$\underline{0.069\pm 0.004}$	0.094±0.005	0.854±0.018	0.061±0.004	12.6±2.5

↓ and ↑ indicate that lower and higher values, respectively, represent better predictive performance. N/A indicates that the residual contribution is not defined for the variant without mechanism decomposition. Bold and underlined values indicate the best and second-best results, respectively, among the compared model variants.

**Table 15:** Performance by terminal-release group (mean ± SD).

Terminal-Release Group	Model	No. Curves	Mean Final Release	Full RMSE ↓	nIAE ↓	Late RMSE ↓	Final-Release MAE ↓	Final-Release Bias
Low 0.60≤R(T)<0.75	FT-Transformer	82	0.683±0.041	0.128±0.007	0.089±0.006	0.139±0.008	0.104±0.011	+0.041±0.014
	Weibull-Param-LightGBM	82	0.683±0.041	0.121±0.006	0.084±0.005	0.129±0.007	0.098±0.010	+0.027±0.012
	SingleRate-NODE	82	0.683±0.041	0.111±0.006	0.075±0.005	0.116±0.006	0.087±0.009	+0.020±0.010
	Fixed Plateau	82	0.683±0.041	0.125±0.008	0.087±0.006	0.145±0.009	0.122±0.013	+0.104±0.015
	MC-NODE	82	0.683±0.041	0.102±0.006	0.067±0.005	0.091±0.006	0.055±0.007	+0.008±0.008
Medium 0.75≤R(T)<0.90	FT-Transformer	134	0.823±0.043	0.109±0.005	0.076±0.004	0.115±0.006	0.085±0.008	+0.012±0.010
	Weibull-Param-LightGBM	134	0.823±0.043	0.104±0.005	0.071±0.004	0.105±0.005	0.078±0.007	+0.004±0.009
	SingleRate-NODE	134	0.823±0.043	0.096±0.005	0.064±0.004	0.096±0.005	0.062±0.006	+0.002±0.007
	Fixed Plateau	134	0.823±0.043	0.104±0.006	0.071±0.005	0.112±0.006	0.080±0.008	+0.061±0.010
	MC-NODE	134	0.823±0.043	0.091±0.004	0.059±0.004	0.086±0.004	0.044±0.006	−0.003±0.006
High R(T)≥0.90	FT-Transformer	105	0.948±0.030	0.105±0.006	0.073±0.005	0.102±0.006	0.078±0.008	−0.006±0.009
	Weibull-Param-LightGBM	105	0.948±0.030	0.101±0.005	0.068±0.004	0.098±0.005	0.066±0.007	−0.003±0.008
	SingleRate-NODE	105	0.948±0.030	0.094±0.005	0.061±0.004	0.091±0.005	0.055±0.006	−0.006±0.007
	Fixed Plateau	105	0.948±0.030	0.087±0.005	0.057±0.004	0.085±0.005	0.039±0.005	+0.016±0.006
	MC-NODE	105	0.948±0.030	0.089±0.005	0.058±0.004	0.084±0.005	0.041±0.005	−0.011±0.006

↓ indicates that a lower value represents better performance. Bold values indicate the best result within each terminal-release group. Late RMSE was calculated over the normalized time interval 0.70≤τ≤1.00. A positive final-release bias indicates overprediction, whereas a negative bias indicates underprediction.

## Data Availability

The original contributions presented in this study are included in the article. Further inquiries can be directed to the corresponding author.
